# The *NRC0* gene cluster of sensor and helper NLR immune receptors is functionally conserved across asterid plants

**DOI:** 10.1093/plcell/koae154

**Published:** 2024-06-04

**Authors:** Toshiyuki Sakai, Mauricio P Contreras, Claudia Martinez-Anaya, Daniel Lüdke, Sophien Kamoun, Chih-Hang Wu, Hiroaki Adachi

**Affiliations:** The Sainsbury Laboratory, University of East Anglia, Norwich Research Park, Norwich NR4 7UH, UK; Laboratory of Crop Evolution, Graduate School of Agriculture, Kyoto University, Mozume, Muko, Kyoto 617-0001, Japan; The Sainsbury Laboratory, University of East Anglia, Norwich Research Park, Norwich NR4 7UH, UK; The Sainsbury Laboratory, University of East Anglia, Norwich Research Park, Norwich NR4 7UH, UK; Instituto de Biotecnología, Universidad Nacional Autónoma de México, Cuernavaca, Morelos 62110, México; The Sainsbury Laboratory, University of East Anglia, Norwich Research Park, Norwich NR4 7UH, UK; The Sainsbury Laboratory, University of East Anglia, Norwich Research Park, Norwich NR4 7UH, UK; The Sainsbury Laboratory, University of East Anglia, Norwich Research Park, Norwich NR4 7UH, UK; Institute of Plant and Microbial Biology, Academia Sinica, Nankang, Taipei 11529, Taiwan; The Sainsbury Laboratory, University of East Anglia, Norwich Research Park, Norwich NR4 7UH, UK; Laboratory of Crop Evolution, Graduate School of Agriculture, Kyoto University, Mozume, Muko, Kyoto 617-0001, Japan; JST-PRESTO, 4-1-8, Honcho, Kawaguchi, Saitama 332-0012, Japan

## Abstract

Nucleotide-binding domain and leucine-rich repeat-containing receptor (NLR) proteins can form complex receptor networks to confer innate immunity. An NLR-REQUIRED FOR CELL DEATH (NRC) is a phylogenetically related node that functions downstream of a massively expanded network of disease resistance proteins that protect against multiple plant pathogens. In this study, we used phylogenomic methods to reconstruct the macroevolution of the NRC family. One of the NRCs, termed *NRC0*, is the only family member shared across asterid plants, leading us to investigate its evolutionary history and genetic organization. In several asterid species, *NRC0* is genetically clustered with other NLRs that are phylogenetically related to NRC-dependent disease resistance genes. This prompted us to hypothesize that the ancestral state of the NRC network is an NLR helper–sensor gene cluster that was present early during asterid evolution. We provide support for this hypothesis by demonstrating that NRC0 is essential for the hypersensitive cell death that is induced by its genetically linked sensor NLR partners in 4 divergent asterid species: tomato (*Solanum lycopersicum*), wild sweet potato (*Ipomoea trifida*), coffee (*Coffea canephora*), and carrot (*Daucus carota*). In addition, activation of a sensor NLR leads to higher-order complex formation of its genetically linked NRC0, similar to other NRCs. Our findings map out contrasting evolutionary dynamics in the macroevolution of the NRC network over the last 125 million years, from a functionally conserved NLR gene cluster to a massive genetically dispersed network.

## Teaser

The *NRC0* gene cluster of sensor and helper immune receptors is functionally conserved across divergent asterid species and predates the massively expanded NLR immune receptor network.

**IN A NUTSHELL**

**Background:** In plants, nucleotide-binding leucine-rich repeat receptors (NLRs) generally exhibit hallmarks of rapid evolution, even at the intraspecific level. While some plant NLRs operate as singletons, many have functionally specialized to recognize pathogen effectors (sensor NLRs) or to activate immune responses (helper NLRs). Interestingly, these functionally specialized NLRs often form gene pairs or clusters in plant genomes and function together in immune activation. In solanaceous plants, the NLR-REQUIRED FOR CELL DEATH (NRC) family genes form complex immune receptor networks composed of genetically dispersed sensor and helper NLRs.

**Question:** Can we trace an evolutionary trajectory of NRC networks evolved across asterid and other Solanaceae-related plant species?

**Findings:** We conducted phylogenomic analyses to reconstruct the macroevolution of the NRC family. Among the NRCs, *NRC0* stands out as the only family member that has remained conserved across asterid species. *NRC0* orthologs often form gene pairs or clusters with sensor NLR subclade genes. We experimentally validated the functional connections within *NRC0* gene clusters for four distantly related asterid species. Our findings revealed that *NRC0* is essential for the hypersensitive cell death response triggered by its genetically linked sensor NLR. In addition, activation of a sensor NLR induces high-order complex formation of its genetically linked NRC0. We propose that *NRC0* emerged early in asterid evolution, and the *NRC0* sensor-helper gene cluster may reflect an ancestral state predating the massively expanded immune receptor networks in the Solanaceae and related asterid species.

**Next steps:** It is crucial to identify the type of pathogen effectors recognized by the NRC0 sensor-helper pairs. Furthermore, elucidating the protein structure of these NRC0 pairs and understanding the determinants of functional specificity between NRC0 sensor-helper proteins are important unanswered questions to map out the evolution of activation mechanisms of plant NLRs.

## Introduction

Plants have evolved an effective innate immune system that is activated by immune receptors upon sensing diverse pathogen molecules in either the extracellular or the intracellular space of host cells. One such immune receptor is the nucleotide-binding domain and leucine-rich repeat (LRR)-containing receptor (NLR) family, composed of intracellular immune receptors recognizing pathogen-secreted proteins called “effectors” (Jones et al. 2016). The evolution of NLRs is marked by a continuous arms race between plants and pathogens, leading to the rapid evolution and diversification of NLR genes even at the intraspecific level (Van de Weyer et al. 2019; Lee and Chae 2020; Prigozhin and Krasileva 2021). NLRs are known as the most diverse protein family in flowering plants, as many plants have hundreds to thousands of diverse NLR genes in their genomes (Shao et al. 2016; Baggs et al. 2017; Kourelis et al. 2021). This diversification and expansion of NLR genes in plants possibly occurred through genome rearrangements such as gene duplication, deletion, and conversion, as well as point mutations and domain insertions (Barragan and Weigel 2021). As a consequence of these events, NLR genes in plant genomes are often found in clusters. For example, in Arabidopsis (*Arabidopsis thaliana*) accessions, 47% to 71% of NLR genes form NLR gene clusters in their genomes (Van de Weyer et al. 2019). Clustered NLR genes tend to have higher nucleotide sequence diversity than nonclustered NLR genes in Arabidopsis (Van de Weyer et al. 2019), thereby providing a reservoir of genetic variation for novel immune specificities against fast-evolving pathogen effectors. Understanding the macroevolutionary dynamics of NLRs across diverse plant species is crucial not only for unraveling the molecular mechanisms underlying plant immunity but also for providing genetic resources of disease resistance traits for global food security.

NLR and NLR-related proteins are key components of innate immunity and nonself recognition not only in plants but also in animals, fungi, and bacteria (Jones et al. 2016; Uehling et al. 2017; Kibby et al. 2023). Plant NLRs have a shared domain architecture of a central nucleotide-binding domain with an APAF-1, various R proteins, a CED-4 (NB-ARC) domain, and a C-terminal LRR domain (Kourelis et al. 2021). At the N-terminal region of plant NLRs, there is a variable domain that can be a coiled-coil (CC) or a toll/interleukin-1 receptor (TIR) domain. Based on the N-terminal domains, plant NLRs are broadly classified into 4 subfamilies: CC-NLRs (CNLs), G10-type CC-NLRs (CC_G10_-NLRs), RESISTANCE TO POWDERY MILDEW 8 (RPW8)-type CC-NLRs (CC_R_-NLRs or RNLs), and TIR-NLRs (TNLs; Lee et al. 2021). Phylogenomic studies revealed that the most widely conserved CC-NLR gene across flowering plant (angiosperm) species is *HOPZ-ACTIVATED RESISTANCE1* (*ZAR1*), which was initially identified in Arabidopsis (Gong et al. 2022; Adachi et al. 2023). As there is no NLR gene genetically clustered to the *ZAR1* locus across angiosperm species, *ZAR1* is defined as a genetic singleton NLR gene throughout its evolution, and indeed, the ZAR1 protein functions as a singleton NLR that does not appear to require other NLRs to activate immunity (Adachi et al. 2023).

Upon effector recognition through its LRR domain and partner receptor-like cytoplasmic kinases, activated ZAR1 forms a homo-pentameric complex called “resistosome,” which functions as a calcium ion (Ca^2+^) channel at the plasma membrane, resulting in induction of the hypersensitive cell death immune response (Wang et al. 2019a, 2019b; Bi et al. 2021). To make a pore on the plasma membrane and cause Ca^2+^ influx, the ZAR1 resistosome exposes a funnel-shaped structure formed by its first α helix (α1 helix) of the N-terminal CC domain (Wang et al. 2019a; Bi et al. 2021). The α1 helix is defined as the “MADA motif” that is conserved in about 20% of CC-NLRs from dicot and monocot species (Adachi et al. 2019a). The MADA sequence is functionally interchangeable between dicot and monocot CC-NLRs, suggesting a conserved immune activation mechanism by MADA-type CC-NLRs across angiosperms. Indeed, upon effector perception, the MADA-type CC-NLR Sr35 in wheat (*Triticum monococcum*) forms the homo-oligomerized resistosome complex and causes Ca^2+^ influx (Förderer et al. 2022).

Although some plant NLRs operate as singletons, many plant NLRs are functionally specialized to recognize pathogen effectors (sensor NLRs) or to activate immune responses (helper NLRs, also known as executor NLRs; Adachi et al. 2019b; Kourelis and Adachi 2022). Interestingly, these functionally specialized NLRs often form gene pairs or clusters in plant genomes and function together in immune activation and regulation. For example, in Arabidopsis, *RESISTANCE TO RALSTONIA SOLANACEARUM1* (*RRS1*)/*RESISTANCE TO PSEUDOMONAS SYRINGAE 4* (*RPS4*) (Narusaka et al. 2009; Le Roux et al. 2015; Sarris et al. 2015), *Chilling Sensitive 1* (*CHS1*)/*Suppressors of chs1-2, 3* (*SOC3*) and *TIR-NB 2* (*TN2*) (Zhang et al. 2017; Liang et al. 2019), *CONSTITUTIVE SHADE-AVOIDANCE 1* (*CSA1*)/*CHILLING SENSITIVE 3* (*CHS3*) (Xu et al. 2015; Yang et al. 2022), and *SUPRESSOR OF NPR1, CONSTITUTIVE 1* (*SNC1*)/*SIDEKICK SNC1 1* (*SIKIC1*), *SIKIC2*, and *SIKIC3* (Dong et al. 2018) form genetically linked pairs or clusters that function together in immune responses. In rice (*Oryza sativa*), *RESISTANCE GENE ANALOG 5* (*RGA5*)/*RGA4* and *PYRICULARIA ORYZAE RESISTANCE K-1* (*Pik-1*)/*Pik-2* are 2 sensor–helper NLR pairs that are found in head-to-head orientation in the genome (Césari et al. 2014; Maqbool et al. 2015; Shimizu et al. 2022; Sugihara et al. 2023).

In addition to NLR pairs, genetically dispersed NLRs often function together and form complex immune receptor networks. In solanaceous plants, CC-NLR proteins known as NLR-REQUIRED FOR CELL DEATH (NRC) function as helper NLRs (NRC-H) for multiple sensor NLRs (NRC-S) to mediate immune responses and to confer disease resistance against diverse pathogens (Wu et al. 2017). Although NRC-H and NRC-S genes are phylogenetically linked and form a hugely expanded NRC superclade, NRC-H and NRC-S genes are scattered throughout the genome of solanaceous plants (Wu et al. 2017). Recent biochemical and cell biology studies revealed that activated NRC-S induces homo-oligomerization of NRC-H, and activated NRC-H forms punctate structures at the plasma membrane (Duggan et al. 2021; Ahn et al. 2023; Contreras et al. 2023a, 2023b). This suggests that in NRC networks, NRC-H proteins activated by NRC-S presumably trigger immune responses at the plasma membrane, as is the case in the ZAR1 resistosome model. Consistent with this view, NRC-H in the NRC superclade has the MADA motif at its N termini (Adachi et al. 2019a). In contrast, the N termini of NRC-S genes have diversified and/or acquired additional extension domains prior to the CC domain (Adachi et al. 2019a; Seong et al. 2020). Based on these findings, a current evolutionary model of the NRC network is that NRC-H and NRC-S evolved from a multifunctional singleton NLR and have functionally specialized into helper and sensor NLRs throughout evolution (Adachi et al. 2019b; Adachi and Kamoun 2022).

In a previous study, the NRC network was proposed to have evolved from a sensor–helper gene cluster (Wu et al. 2017). Outside of the asterid lineages, the Caryophyllales species sugar beet (*Beta vulgaris*) genome encodes 1 NRC-H and 2 NRC-S genes that form a gene cluster (Wu et al. 2017). Therefore, the NRC superclade presumably emerged from a pair of genetically linked NLRs about 100 million years ago (mya) before the asterids and Caryophyllales lineages split. However, our knowledge of how NRC networks evolved across asterid and other Solanaceae-related plant species remains limited. In particular, the evolutionary dynamics of the NRC-Hs across the asterids have not been studied in detail. Here, we show that *NRC0* is the only NRC-H family member that has remained conserved across asterid species. *NRC0* orthologs form gene pairs or clusters with gene(s) from the NRC-S clade and are widely distributed in Cornales, campanulids, and lamiids. We experimentally validated the functional connections between NRC0 and genetically linked NRC-S (NRC0-S) in the *Nicotiana benthamiana* model system. Furthermore, activation of a tomato NRC0-S resulted in high-order complex formation of its genetically linked NRC0 similar to the oligomerization observed for other NRCs (Ahn et al. 2023; Contreras et al. 2023a, 2023b). We propose that the *NRC0* sensor–helper gene cluster may reflect an ancestral state of the NRC network; *NRC0* emerged early in asterid evolution and has massively expanded into immune receptor networks in the Solanaceae and related asterid species. This study highlights contrasting evolutionary dynamics between the genetically and the functionally linked *NRC0* gene cluster and the massively expanded and genetically dispersed NRC network of lamiid plants.

## Results

### *NRC0* is the most conserved NRC-H gene in asterids

**Figure 1.**
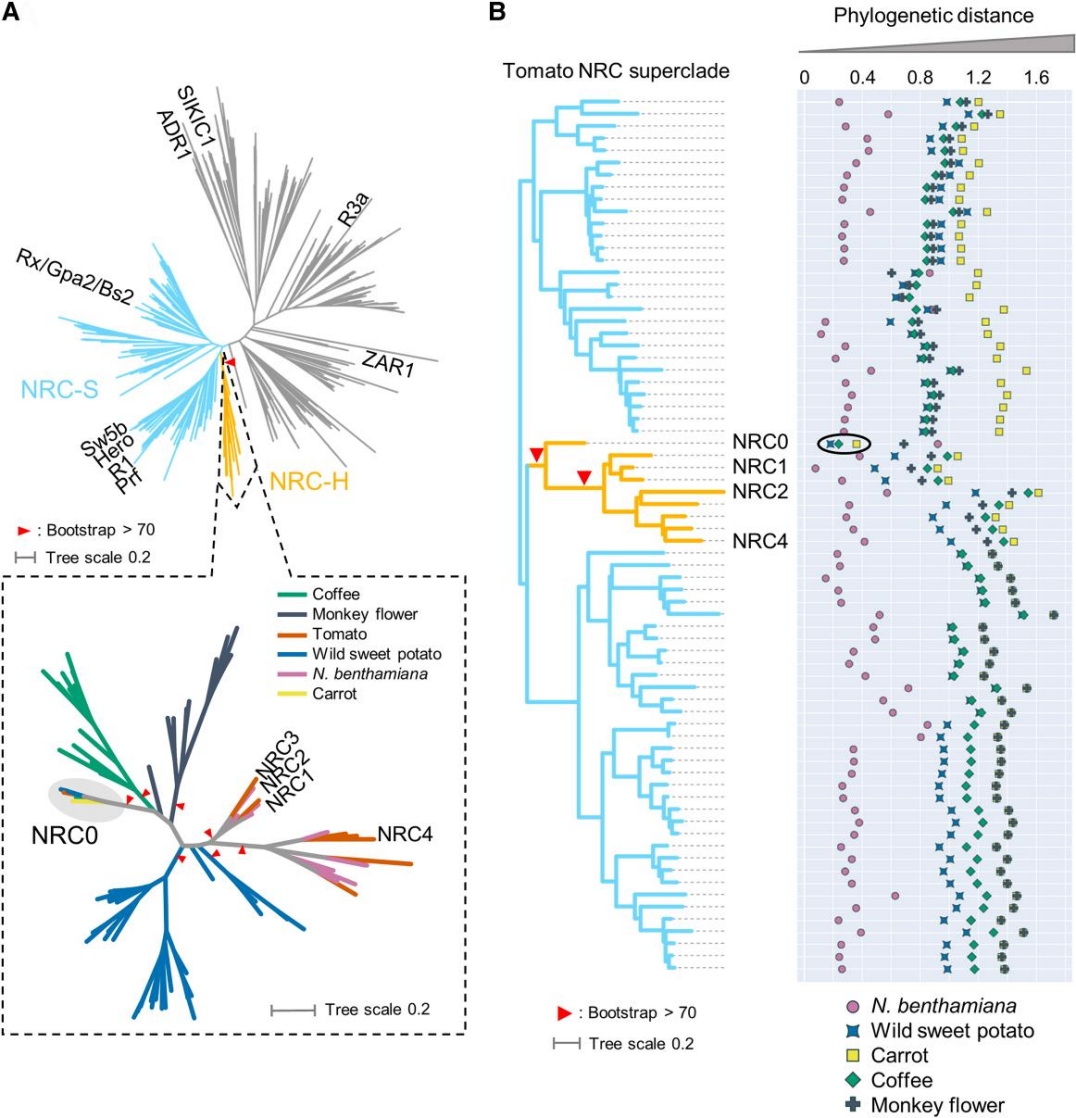
*NRC0* is the most conserved helper clade NRC in asterids. **A)** A phylogeny of NLRs identified from asterids (carrot, monkey flower, coffee, wild sweet potato, *N. benthamiana*, and tomato). The phylogenetic unrooted tree was generated in RAxML version 8.2.12 with the Jones-Taylor-Thornton (JTT) model using NB-ARC domain sequences of 1,661 NLRs identified from carrot, monkey flower, coffee, wild sweet potato, *N. benthamiana*, and tomato reference genome by using the NLRtracker and 39 functionally validated NLRs. The scale bar indicates the evolutionary distance in amino acid substitution per site. In the top left phylogenetic tree, the NRC superclades are described with different branch color codes. The bottom left phylogenetic tree describes the NRC-H subclade with different color codes based on plant species. The red arrow heads indicate bootstrap support >0.7 and are shown for the relevant nodes. **B)** The phylogenetic distance of 2 NRC-H and NRC-S nodes between tomato and other plant species. The phylogenetic distance was calculated from the NB-ARC phylogenetic tree shown in **A**. The closest distances are plotted with different colors based on plant species in the same way as **A**. Representative tomato NRC-Hs are highlighted. The most conserved NRC-H is highlighted by a black oval.

We hypothesized that the most conserved NRC gene across asterid species is most likely to reflect an ancestral state of the expanded NRC networks. To determine the distribution of NRC-H genes across plant species, we first annotated NLR genes from reference genome databases of 6 representative plant species from asterids: carrot (*Daucus carota*, DCAR-), monkey flower (*Mimulus guttatus*, Migut-), coffee (*Coffea canephora*, Cc-), wild sweet potato (*Ipomoea trifida*, itf-), *N. benthamiana* (NbS-), and tomato (*Solanum lycopersicum*, Solyc-) by using the NLRtracker pipeline (Kourelis et al. 2021; Supplementary File S1). To classify NRC superclade genes from the asterid NLRome dataset, we performed a phylogenetic analysis using the NB-ARC domain sequences of 1,661 annotated NLRs and 39 functionally validated NLRs (Fig. 1A). In total, we identified 83 NRC-H genes from 6 plant species (1 gene from carrot; 11 genes from monkey flower; 13 genes from coffee; 36 genes from wild sweet potato; 13 genes from *N. benthamiana*; and 9 genes from tomato; Supplementary Fig. S1). While most of the NRC-H forms plant-lineage-specific subclades or clusters with previously defined Solanaceae (*N. benthamiana* and tomato) NRC-H subclade, there is 1 unique NRC-H subclade containing NRC-H sequences derived from 4 different plant species, carrot, coffee, wild sweet potato, and tomato (Fig. 1A; Supplementary Fig. S1). We named the subclade NRC0, with each of the 4 species having 1 or 2 *NRC0* orthologous genes (DCAR_023561, Cc11_g06560, itf14g00240, itf14g00270, Solyc10g008220; Supplementary Data Set 1). In contrast to the 4 species, *NRC0* is not found in monkey flower and *N. benthamiana*.

To further evaluate *NRC0* conservation relative to other NRC-H, we used a phylogenetic tree of 805 NLR genes including NRC-H and NRC-S from the 6 asterid species (carrot, monkey flower, coffee, wild sweet potato, *N. benthamiana*, and tomato) and 9 functionally validated NRCs to calculate the phylogenetic (patristic) distance between each of the 72 tomato NRC-H/-S and their closest neighbor gene from each of the other plant species. We found that NRC0 displays the shortest patristic distance to its orthologs compared with other NRCs (Fig. 1B). These phylogenetic analyses suggest that *NRC0* is possibly the most widely conserved NRC-H gene in asterids.

### Comparative analyses of NLR genes across asterid genomes identify a conserved *NRC0* gene cluster of candidate sensors and helpers

Plant sensor and helper NLRs often function in genetically linked pairs, while solanaceous NRCs such as NRC2, NRC3, and NRC4 form phylogenetically related but genetically dispersed NLR networks (Wu et al. 2017). To determine the degree to which helper NLRs, including *NRC0*, form NLR gene clusters in the genome, we conducted a gene cluster analysis of whole NLRomes annotated from 4 asterid species, carrot, coffee, wild sweet potato, and tomato. In this analysis, we extracted genetically linked NLRs that have genetic distances <50 kb. The gene cluster information was mapped onto the NLR phylogenetic tree of the 4 plant species. In total, we found 1,146 genetically linked gene pairs in NLRomes of the 4 plant species (141 gene clusters in carrot, 324 gene clusters in coffee, 536 gene clusters in wild sweet potato, and 145 gene clusters in tomato; Supplementary Fig. S2 and Data Sets 2 and 3).

**Figure 2.**
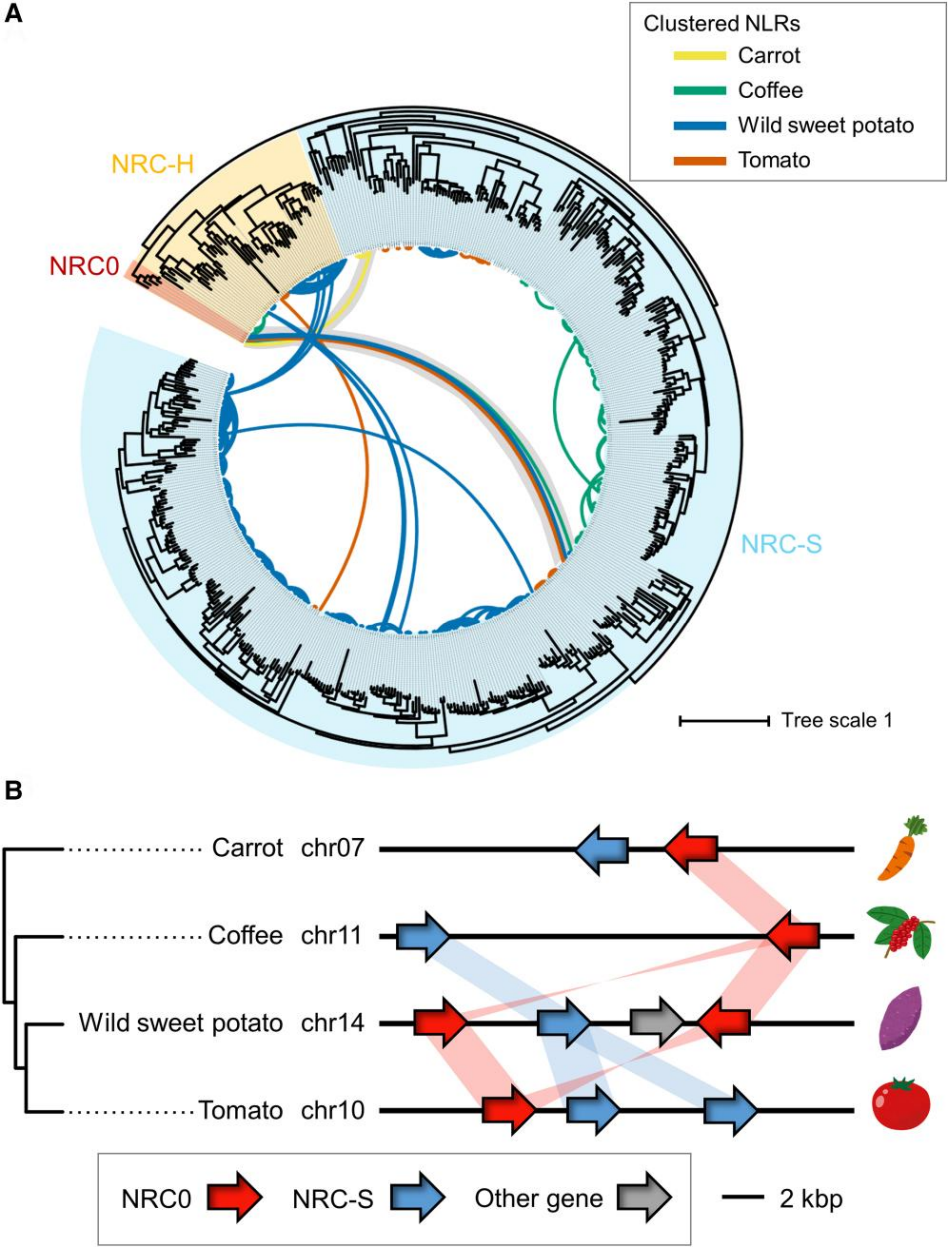
*NRC0* forms a conserved gene cluster with members of the NRC-S clade in asterids. **A)** A phylogeny of NRC family genes from carrot, coffee, wild sweet potato, and tomato with NLR gene cluster information. The phylogenetic unrooted tree was generated in RAxML version 8.2.12 with the JTT model using NB-ARC domain sequences of 513 NRCs identified in Fig. 1. The scale bar indicates the evolutionary distance in amino acid substitution per site. The NRC subclades are described with different background colors. The connected lines between nodes indicate genetically linked NLRs (distance < 50 kb) with different colors based on plant species. The genetic link between *NRC0* and *NRC-S* is highlighted. **B)** A schematic representation of *NRC0* loci in carrot, coffee, wild sweet potato, and tomato. The red, blue, and gray arrows indicate *NRC0*, *NRC-S* genetically linked with *NRC0*, and other genes, respectively. The red and blue bands indicate phylogenetically related genes.

In the NRC superclade, 438 genetically linked gene pairs exist in the genomes (3 gene clusters in carrot, 45 gene clusters in coffee, 347 gene clusters in wild sweet potato, and 43 gene clusters in tomato; Fig. 2A; Supplementary Data Sets 2 and 3). Out of 438 gene pairs, 378 are NRC-S gene pairs and 47 are NRC-H gene pairs. Notably, there are 13 genetically linked gene pairs that consist of both NRC-H and NRC-S genes (Fig. 2A). Among them, only 6 gene pairs are conserved in the 4 plant species and are formed by *NRC0* and NRC-S clade genes (DCAR_023560 and DCAR_023561, Cc11_g06550 and Cc11_g06560, itf14g00240 and itf14g00250, itf14g00250 and itf14g00270, Solyc10g008220 and Solyc10g008230, Solyc10g008220 and Solyc10g008240) (Fig. 2, A and B). Although we found 7 additional gene clusters (6 gene clusters in wild sweet potato, 1 gene cluster in tomato), these gene clusters appeared to be plant lineage specific (Fig. 2A; Supplementary Data Set 2). CC_R_-NLRs, *ACTIVATED DISEASE RESISTANCE 1* (*ADR1*) and *N REQUIREMENT GENE 1* (*NRG1*), are also known as conserved helper subfamily genes (Shao et al. 2016; Baggs et al. 2020; Liu et al. 2021). We noted that *ADR1* and *NRG1* in carrot, coffee, wild sweet potato, and tomato form gene clusters with their paralogs but not with genes from other NLR subfamilies (Supplementary Fig. S2 and Data Sets 2 and 3). Taken together, these findings suggest that *NRC0* stands out as a widely conserved gene cluster of candidate helper–sensor NLRs in asterids.

### The *NRC0* gene cluster predates the NRC expansion in lamiids

**Figure 3.**
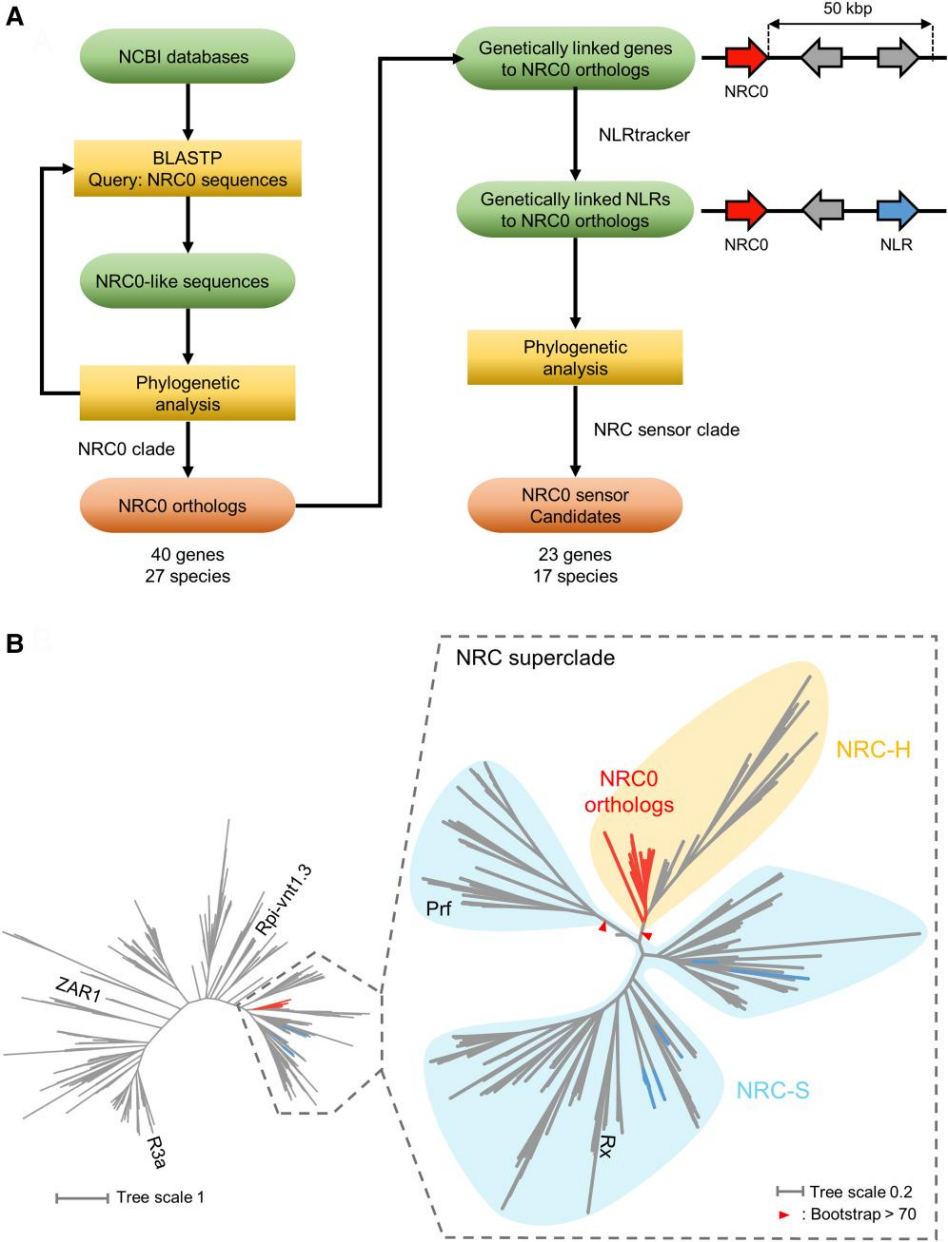
Phylogenomic analyses identify 40 *NRC0* orthologs from 27 asterid species that are linked to 23 *NRC0-S* in 17 species. **A)** A workflow for computational analyses in searching *NRC0* orthologs and *NRC0-S* candidates. TBLASTN/BLASTP searches and subsequent phylogenetic analyses were performed to identify *NRC0* orthologs from plant genome/proteome datasets. We extracted *NRC0-S* candidates by performing a gene cluster analysis, using the NLRtracker (Kourelis et al. 2021), and conducting a phylogenetic analysis. **B)***NRC0* orthologs exist in a subclade of the NRC-H clade. The phylogenetic unrooted tree was generated in RAxML version 8.2.12 with the JTT model using NB-ARC domain sequences of NRC0, NRC0-S, 15 functionally validated CC-NLRs, and 1,194 CC-NLRs identified from 6 representative asterids: *Ny. sinensis*, *Cam. sinensis*, *Cy. cardunculus*, *D. carota*, *Se. indicum*, and *S. lycopersicum*. The scale bar indicates the evolutionary distance in amino acid substitution per site. The red and blue branches indicate NRC0 and NRC0-S, respectively. The NRC subclades are described with different background colors. The red arrow heads indicate bootstrap support >0.7 and are shown for the relevant nodes.

To further examine the distribution of the *NRC0* gene cluster across plant species, we searched for *NRC0* orthologs by running a 2-stage computational pipeline based on iterated BLAST searches of plant genome and protein databases and phylogenetic analysis (Fig. 3A). First, we defined NRC-H genes as *NRC0* orthologs if the NRC-H genes belong to a phylogenetically well-supported clade with *NRC0* from carrot, coffee, wild sweet potato, and tomato (Fig. 3, A and B). Based on this definition, we identified 40 *NRC0* orthologs from 27 asterid species that classified in the NRC0 phylogenetic subclade (Fig. 3, A and B; Supplementary Data Set 1). In the second stage, we applied gene cluster analysis with a cutoff (genetic distances <50 kb) to identify NLR genes that are genetically linked to the obtained *NRC0* orthologs (Fig. 3A). The 50 kb distance cutoff between adjacent NLRs was set by following the gene cluster analysis of Arabidopsis NLRome (Lee and Chae 2020). Among the 40 *NRC0* ortholog genes, 20 *NRC0* genes from 17 species are genetically linked with 23 NLR genes in NRC-S subclades (Fig. 3, A and B).

**Figure 4.**
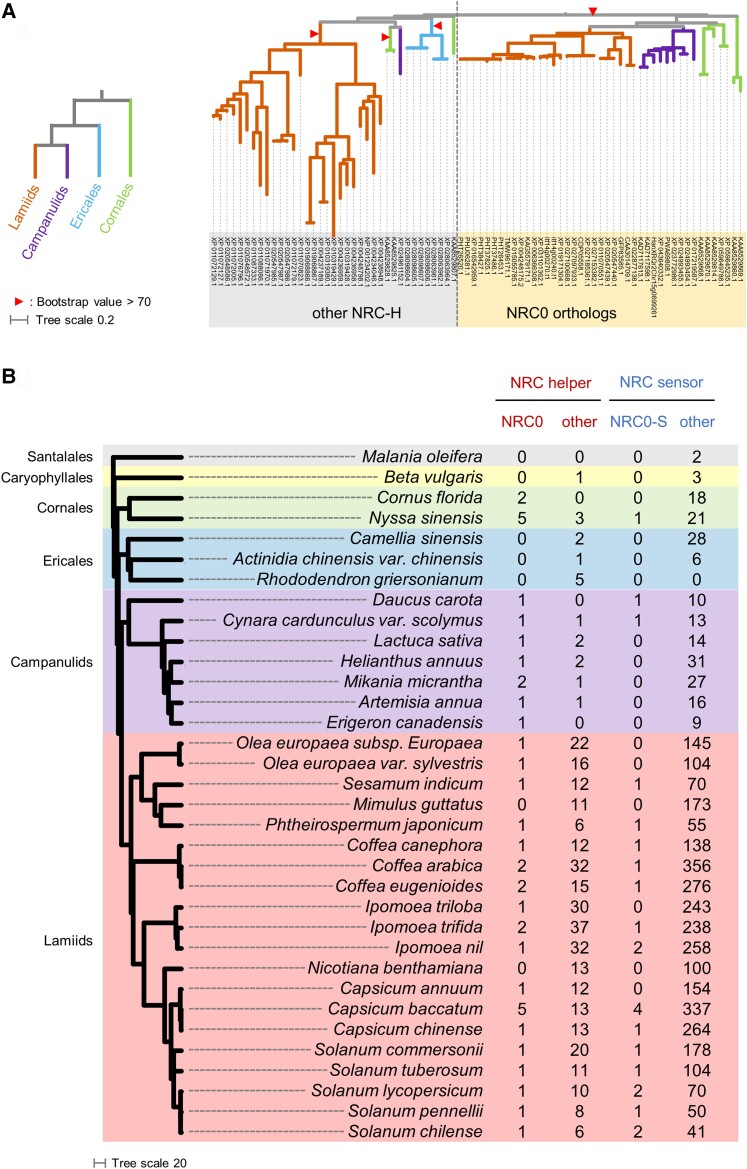
The *NRC0* gene cluster predates the massively expanded NRC network of lamiids. **A)** A phylogeny of NRC-H subfamily defines *NRC0* orthologs and other NRCs. The phylogenetic unrooted tree was generated in RAxML version 8.2.12 with the JTT model using full-length amino acid sequences of 80 NRC-Hs. The scale bar indicates the evolutionary distance in amino acid substitution per site. The phylogenetically well-supported clade (bootstrap value >70) containing NRC0 from Cornales, campanulids, and lamiids is defined as the NRC0 subclade. **B)** Distribution of the number of *NRC* genes across asterids. The left phylogenetic tree of plant species was extracted from a previous study (Smith and Brown 2018). The scale bar indicates branch length in million years ago. The columns on the right indicate the number of *NRC0*, other *NRC-H* and *NRC0-S* genes, and other *NRC-S* genes from 32 asterid, 1 Caryophyllales, and 1 Santalales species. In phylogenetic trees, the branch (**A**) and background (**B**) colors indicate plant orders.

Based on our criteria for the *NRC0* phylogenetic and genetic cluster, *NRC0* orthologs and their genetically linked NLRs (referred to as NRC0-dependent sensor candidates: NRC0-S) are widely distributed in asterids, Cornales, campanulids, and lamiids but not found in Ericales (Fig. 4A; Supplementary Data Set 1). Overall, these results suggest that the *NRC0* gene cluster emerged early in the asterid lineage.

In addition to the gene distribution analysis of *NRC0*, we investigated the number of other NRC-H and NRC-S genes across asterid species. We used NLRtracker to annotate NLR genes from 32 asterid, 1 Caryophyllales, and 1 Santalales species, and classified NRC-H and NRC-S genes based on phylogenetic analysis. We found NRC-H and NRC-S are drastically expanded in lamiids, compared with other plant orders in asterids (Fig. 4B; Supplementary Data Set 4). In Cornales, Ericales, and campanulids, the number of NRC-H genes ranges from 1 to 8 and that of NRC-S genes ranges from 0 to 31 across species in annotations and gene definitions used (Fig. 4B; Supplementary Data Set 4). Notably, a Cornales species, *Cornus florida*, has 2 *NRC0* genes (XP_059649788.1 and XP_059645235.1), but no other NRC-H (Fig. 4B). Although the *Cor. florida NRC0* genes are not genetically clustered with NRC-S genes within <50 kb genetic distance, 8 and 4 NRC-S genes are located on the same scaffolds with XP_059649788.1 and XP_059645235.1, respectively (Supplementary Fig. S3). In lamiids, the number of NRC-H and NRC-S genes ranges from 7 to 39 and from 43 to 357, respectively (Fig. 4B; Supplementary Data Set 4). Taken together, the expansion of NRC genes occurred primarily in lamiid species, but not in other asterid plants.

### *NRC0* orthologs carry the N-terminal sequence pattern required for cell death responses

**Figure 5.**
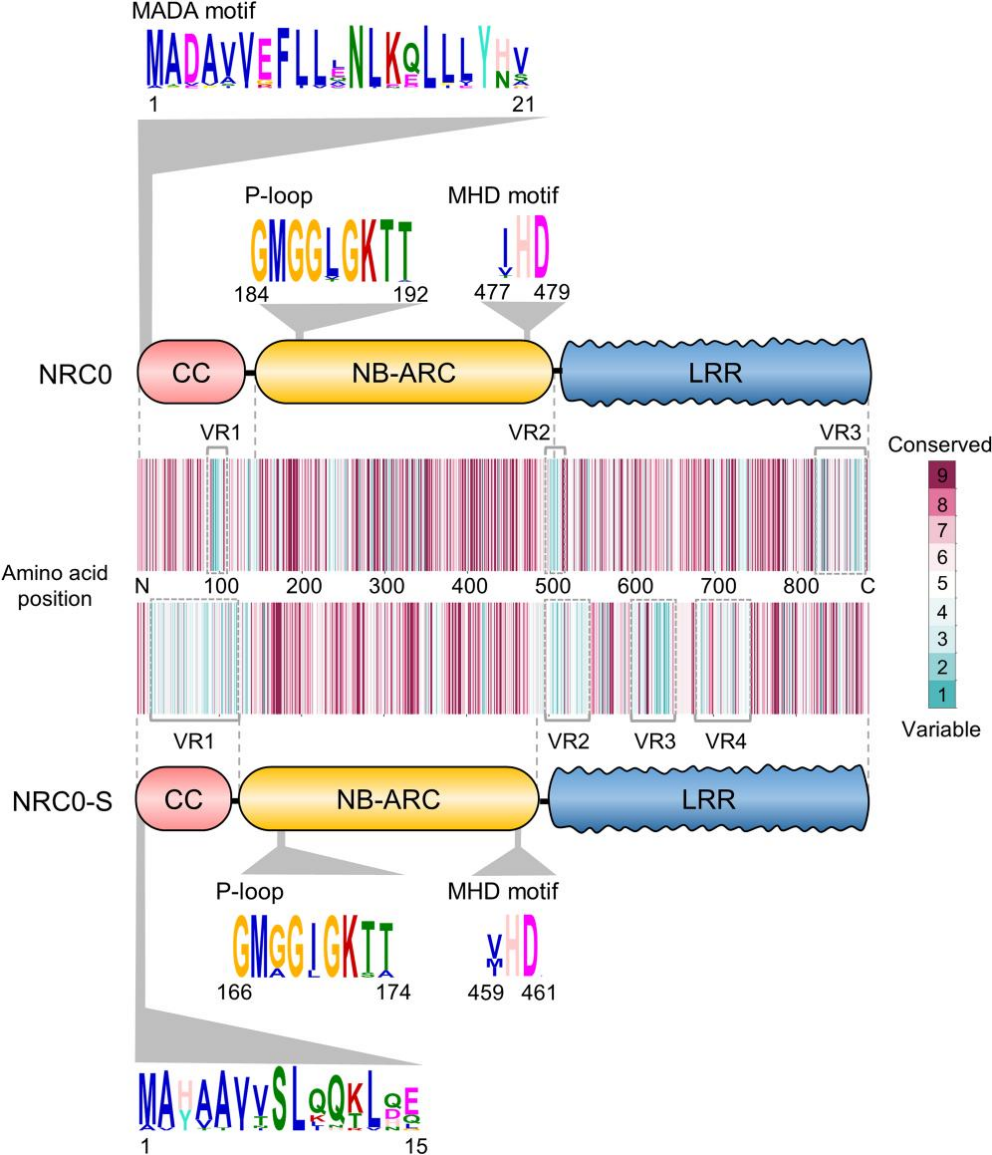
NRC0 orthologs, but not their genetically linked sensors, carry the N-terminal MADA motif required for hypersensitive cell death response. A schematic representation of conserved sequence patterns across NRC0 orthologs and NRC0 sensor candidates (NRC0-S). Consensus sequence patterns were identified by using MEME with amino acid sequences of 40 NRC0 orthologs and 23 NRC0-S, respectively. Conservation and variation of each amino acid among NRC0 orthologs and NRC0-S were calculated based on amino acid alignment via the ConSurf server (https://consurf.tau.ac.il). The conservation scores were mapped onto each amino acid position in tomato NRC0 (XP_004248175.2) and tomato NRC0-S (XP_004248174.1).

In the Solanaceae NRC network, the MADA motif remains at the very N terminus of NRC-H, whereas NRC-S has distinct sequences at its N-terminal region (Adachi et al. 2019a). Therefore, we hypothesized that NRC0 orthologs carry the MADA motif at their N termini for induction of cell death responses. To test this, we first ran MEME (Multiple EM for Motif Elicitation; Bailey and Elkan 1994) to search for conserved sequence patterns among the 40 NRC0 orthologs and 23 NRC0-S. All NRC0 and 10 NRC0-S carry typical CC-NB-LRR domain architecture, while 13 NRC0-S lack either the CC or the LRR domain (Supplementary Data Set 1). None of the NRC0 or NRC0-S was annotated with integrated domains that can be found in sensor NLRs for effector recognition (Fig. 5; Supplementary Data Set 1).

A MEME analysis revealed 20 conserved sequence motifs that span across the NRC0 orthologs and 20 conserved sequence motifs that span across NRC0-S (Fig. 5; Supplementary Tables S1 and S2). Within the MEME motifs, 5th and 13th MEME in the NB-ARC domain of NRC0 and 5th and 11th MEME in the NB-ARC domain of NRC0-S match the p-loop and MHD motifs that coordinate binding and hydrolysis of ATP (Fig. 5). Notably, in NRC0 and NRC0-S, we detected a MEME motif that is positioned at the very N terminus where the MADA motif is generally found (Fig. 5). Next, we used the HMMER software (Eddy 1998) to query the NRC0 orthologs and NRC0-S with a previously developed MADA motif–hidden Markov model (MADA-HMM; Adachi et al. 2019a). This HMMER search detected the MADA motif in 90% (36/40) of NRC0 orthologs but in none of the NRC0-S (0/23) (Supplementary Data Set 1). Indeed, the sequence patterns of the N termini are different between NRC0 and NRC0-S. Instead of the MADA sequence, NRC0-S members have the “MAHAAVVSLxQKLxx” sequence at their N termini (Fig. 5).

To further investigate sequence conservation and variation among NRC0 and NRC0-S, we used ConSurf (Ashkenazy et al. 2016) to calculate a conservation score for each amino acid and generate a diversity barcode for NRC0 and NRC0-S, respectively (Fig. 5). NRC0 orthologs have highly conserved amino acid sequences across their entire domains and display a few variable regions (VRs) at the middle of the CC domain (VR1), junction of the NB-ARC and LRR domains (VR2), and C-terminal end of the LRR domain (VR3; Fig. 5). In the case of NRC0-S, the amino acid sequence is variable in the CC and LRR domains (VR1 ∼ VR4), although the NB-ARC domain sequence is highly conserved across NRC0-S members (Fig. 5).

Taken together, NRC0 orthologs carry highly conserved sequences throughout the full protein and display a canonical N-terminal MADA motif. In contrast, NRC0-S sequences tend to be more variable especially in the CC and in parts of the LRR domains and lack a typical MADA motif.

### NRC0 are helper NLRs functionally connected with their genetically linked NLR sensors

**Figure 6.**
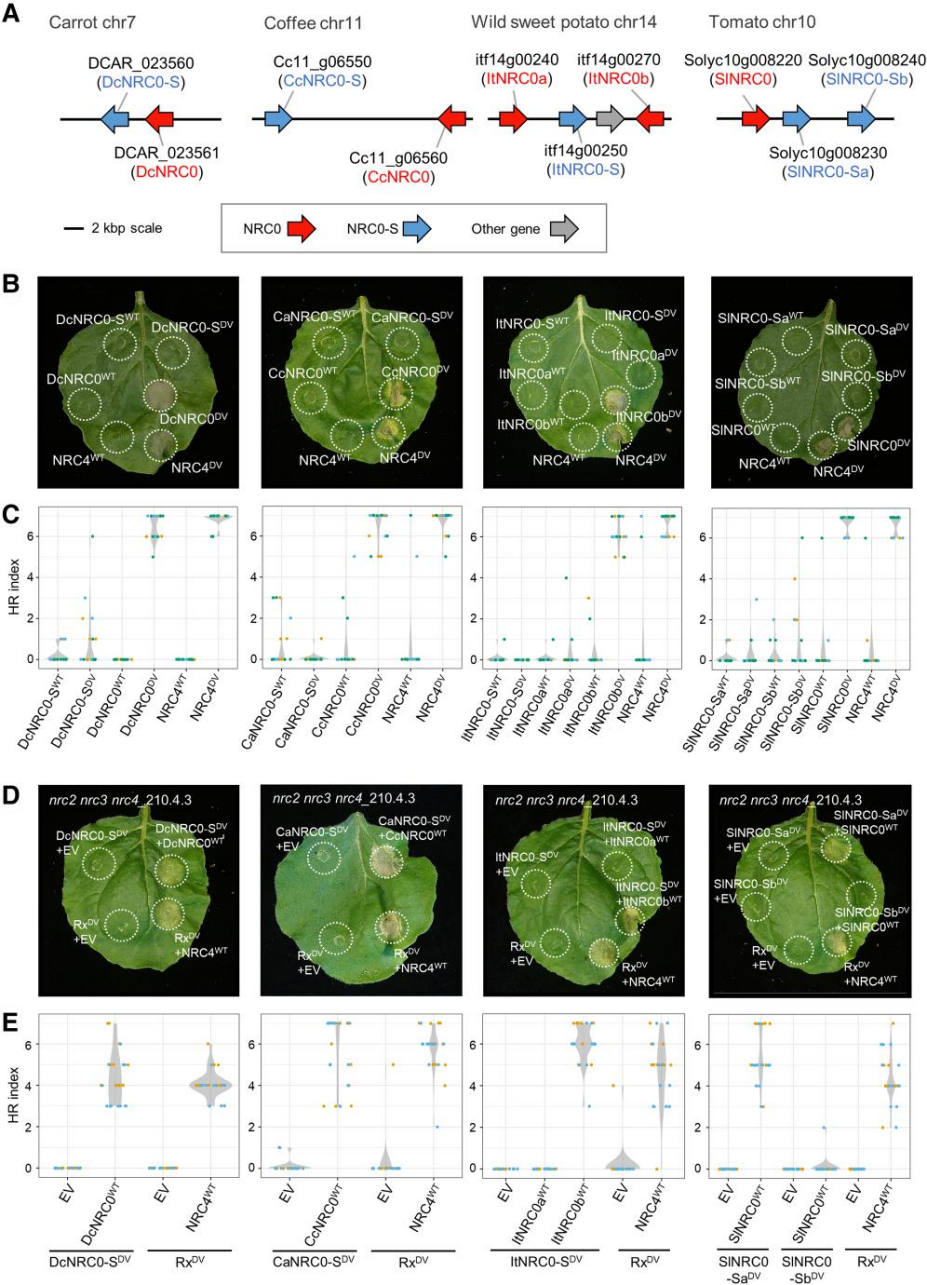
NRC0 is required for the genetically linked NRC-S to trigger the hypersensitive cell death response in *N. benthamiana*. **A)** A schematic representation of *NRC0* loci in carrot, coffee, wild sweet potato, and tomato. **B)** Wild-type NRC0, NRC0-S, NRC4, and the MHD mutants were expressed in *N. benthamiana* leaves by agroinfiltration. Cell death phenotype was recorded 5 d after the agroinfiltration. **C)** Violin plots showing cell death intensity scored as an hypersensitive response (HR) index based on 18 replicates (different leaves from independent plants) in 3 independent experiments of **B**. Each experiment is visualized with different dot colors. **D)** Representative images of autoactive cell death after a coexpression of wild-type NRC0 (NRC0^WT^) and MHD mutants of the NRC0 sensor (NRC0-S^DV^) in the *N. benthamiana nrc2 nrc3 nrc4* mutant line. Empty vector (EV), wild-type NRC4 (NRC4^WT^) and the MHD mutant of sensor Rx (Rx^DV^) were used as controls. Photographs were taken at 5 d after agroinfiltration. **E)** Violin plots showing cell death intensity scored as an HR index based on 12 replicates (different leaves from independent plants) in 2 independent experiments of **D**. Each experiment is visualized with different dot colors.

Since NRC0, but not NRC0-S, carries the MADA motif at its N termini, we hypothesized that NRC0 functions as a helper and NRC0-S relies on its genetic partner NRC0 to trigger immune responses. To experimentally validate this hypothesis, we first cloned *NRC0* and *NRC0-S* genes from carrot (DcNRC0: DCAR_023561, DcNRC0-S: DCAR_023560), coffee (CcNRC0: Cc11_g06560, CaNRC0-S: XM_027242939.1 in *Coffea arabica*, orthologous gene to Cc11_g06550), wild sweet potato (ItNRC0a: itf14g00240, ItNRC0b: itf14g00270, ItNRC0-S: itf14g00250), and tomato (SlNRC0: Solyc10g008220, SlNRC0-Sa: Solyc10g008230, SlNRC0-Sb: Solyc10g008240) as wild-type sequences (referred to as NRC0^WT^ or NRC0-S^WT^; Fig. 6A). We introduced an aspartic acid (D) to valine (V) mutation in the MHD motif to generate autoactive mutants of each NLR (referred to as NRC0^DV^ or NRC0-S^DV^) and tested the autoactive cell death activity in *N. benthamiana*, a species that does not have NRC0 in its genome (Fig. 4B).

Interestingly, 4 out of 5 tested NRC0, DcNRC0, CcNRC0, ItNRC0b, and SlNRC0 caused macroscopic cell death in *N*. *benthamiana* leaves when expressed as MHD mutants, but the NRC0-S did not (Fig. 6, B and C). As a control, we expressed the *N. benthamiana* NRC-H NRC4 MHD mutant NRC4^D478V^ (referred to as NRC4^DV^) that causes autoactive cell death (Adachi et al. 2019a; Fig. 6, B and C). Although we occasionally observed weak cell death by expressing DcNRC0-S^DV^ and SlNRC0-Sb^DV^, there was no visible cell death when expressing the majority of the DcNRC0-S^DV^ and SlNRC0-Sb^DV^ constructs, similar to the other NRC0-S (Fig. 6, B and C). This result suggests that the MADA-type CC-NLR NRC0 but not NRC0-S has the capacity to trigger hypersensitive cell death by itself.

Our observation that *NRC0-S* genes are genetically clustered with helper *NRC0* genes prompted us to determine whether NRC0-S functionally connects with NRC0. To test this, we expressed NRC0-S MHD mutants with or without their genetically linked wild-type NRC0 in the *nrc2 nrc3 nrc4* knockout *N. benthamiana* line. Notably, we observed that some NRC0-S MHD mutants showed macroscopic cell death in the presence of their genetically linked NRC0 (Fig. 6, D and E). For instance, a coexpression of DcNRC0-S^DV^ and DcNRC0^WT^, CaNRC0-S^DV^ and CcNRC0^WT^, ItNRC0-S^DV^ and ItNRC0b^WT^, SlNRC0-Sa^DV^ and SlNRC0^WT^ triggered a cell death response (Fig. 6, D and E). In this experiment, Rx was used as a control of NRC-dependent sensor NLR functioning with NRC-H NRC2, NRC3, and NRC4 (Wu et al. 2017). NRC4 expression complemented the cell death response triggered by an autoactive MHD mutant of Rx (Rx^D460V^; Bendahmane et al. 2002) in the *nrc2 nrc3 nrc4* knockout line (Fig. 6, D and E).

To further determine whether the NRC0/NRC0-S pair functions with other sensor or helper NLRs in the cell death response, we first validated functional connections between SlNRC0 and 6 representative NRC-S, Gpa2, and Prf (NRC2/NRC3-dependent sensor NLRs), R1 and Rpi-blb2 (NRC4-dependent sensor NLRs), and Rx and Sw-5b (NRC2/NRC3/NRC4-dependent sensor NLRs; Wu et al. 2017) in the *N. benthamiana nrc2 nrc3 nrc4* knockout line. To activate the sensor NLRs, we coexpressed cognate effector genes with avirulence (AVR) activity or used the autoactive MHD mutant of Sw-5b (Sw-5b^D857V^; Derevnina et al. 2021). In contrast to SlNRC0-S, none of the 6 sensor NLRs signal through SlNRC0 to trigger significant cell death response (Supplementary Fig. S4; Huang et al. 2023). SlNRC3 and SlNRC4 were used as controls and complemented cell death response triggered by Gpa2, Prf, Rx, and Sw-5b^D857V^, and R1 and Rpi-blb2, respectively (Supplementary Fig. S4). In this experiment, we observed mild autoactive cell death when Gpa2/SlNRC3 and Rx/SlNRC3 pairs were coexpressed in the absence of cognate AVRs (Supplementary Fig. S4, A, B, E, and F).

Next, we tested 9 tomato NRCs, SlNRC1, SlNRC2, SlNRC3, SlNRC4a, SlNRC4b, SlNRC4c, SlNRC5, SlNRC6, and SlNRC7, in the coexpression experiment with SlNRC0-S^DV^. *SlNRC0-Sa*, *SlNRC0*, and 6 other NRCs (*SlNRC1*, *SlNRC2*, *SlNRC3*, *SlNRC4a*, *SlNRC4b*, and *SlNRC7*) were expressed in both tomato leaf and root tissues, while *SlNRC4c*, *SlNRC5*, and *SlNRC6* were highly expressed in tomato roots (Supplementary Fig. S5A). We observed that none of the tested tomato NRC-H facilitated SlNRC0-S^DV^ in the cell death response (Supplementary Fig. S5, B, C, and F). In the control experiment, SlNRC1, SlNRC2, SlNRC3, SlNRC4a, and SlNRC4b complemented the Rx-mediated cell death (Supplementary Fig. S5, D to F; Lüdke et al. 2023).

Taken together, our results indicate that NRC0-S requires the genetically linked NRC0 to trigger an immune response. This sensor–helper functional connection is specific to the *NRC0* cluster genes, and this pairing is not connected to other sensor and helper nodes in the massively expanded Solanaceae NRC network.

### Coexpression of mismatched NRC0 and NRC0-dependent sensor pairs from different asterid species reveals evolutionary divergence

Our finding that the *NRC0* cluster is conserved in asterid species suggests that NRC0 and NRC0-S are functionally paired across asterids. However, the degree to which coevolution between sensors and helpers has resulted in functional incompatibilities over evolutionary time is unclear. We explored whether sensor–helper pairs have functionally diverged over evolutionary time by coexpressing mismatched pairs from different species, i.e. MHD mutants of each NRC0-S with wild-type NRC0 from 4 asterid species (carrot, coffee, wild sweet potato, and tomato) in the *nrc2 nrc3 nrc4 N. benthamiana* mutant line.

**Figure 7.**
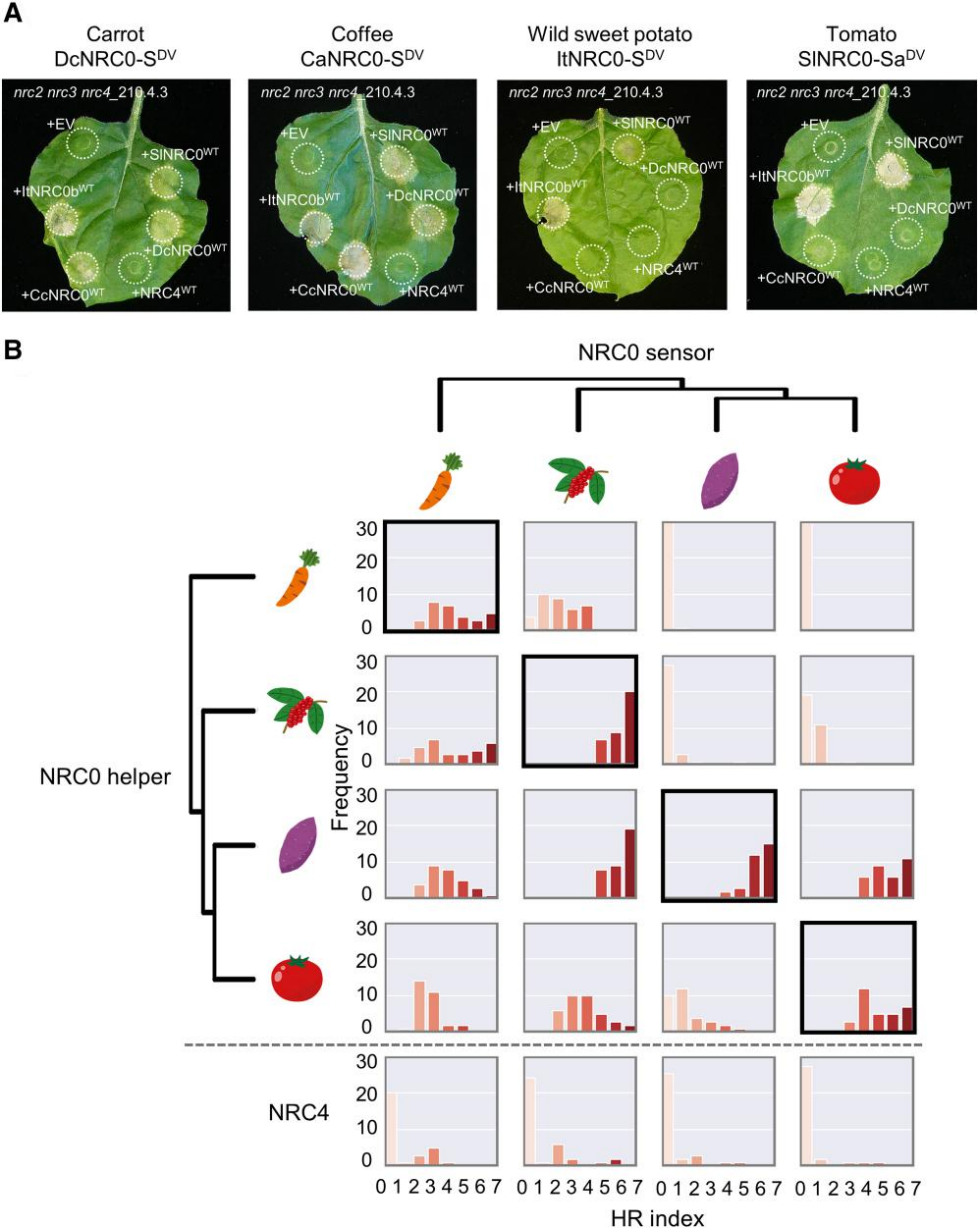
NRC0 sensors have different compatibilities in inducing the hypersensitive cell death with NRC0 orthologs from across asterids. **A)** The photographs show representative images of autoactive cell death after a coexpression of MHD mutants of the NRC0 sensor (NRC0-S^DV^) with wild-type NRC0 (NRC0^WT^) from 4 asterid species (carrot, coffee, wild sweet potato, and tomato) in the *N. benthamiana nrc2 nrc3 nrc4* mutant line. EV and *N. benthamiana* wild-type NRC4 (NRC4^WT^) were used as controls. Photographs were taken at 5 d after agroinfiltration. **B)** A matrix showing the cell death response triggered by NRC0 and NRC0-S^DV^. The histograms describe cell death intensity scored in Supplementary Fig. S6.

We observed functional connections between NRC0-S and NRC0 across asterids with different specificities (Fig. 7, A and B; Supplementary Fig. S6). For instance, a coexpression of either DcNRC0-S^DV^ or CaNRC0-S^DV^ and 4 tested NRC0, DcNRC0^WT^, CcNRC0^WT^, ItNRC0b^WT^, or SlNRC0^WT^ triggered cell death responses (Fig. 7, A and B; Supplementary Fig. S6). Unlike carrot and coffee NRC0-S, ItNRC0-S^DV^ and SlNRC0-Sa^DV^ triggered cell death responses only with ItNRC0b^WT^ or SlNRC0^WT^ (Fig. 7, A and B; Supplementary Fig. S6). As a control, we coexpressed *N. benthamiana* NRC4^WT^ with DcNRC0-S^DV^, CaNRC0-S^DV^, ItNRC0-S^DV^, and SlNRC0-Sa^DV^ and 67% to 84% of the tested samples did not show macroscopic cell death response (Fig. 7, A and B; Supplementary Fig. S6). Taken together, NRC0-S in carrot, a species of campanulids, and coffee, a sister lineage of lamiids, showed functional connections across all of the tested NRC0, while NRC0-S in wild sweet potato and tomato was specifically functional only with NRC0 from Solanales.

### Activated NRC0-dependent sensor leads to high-order complex formation of its genetically linked helper NRC0

**Figure 8.**
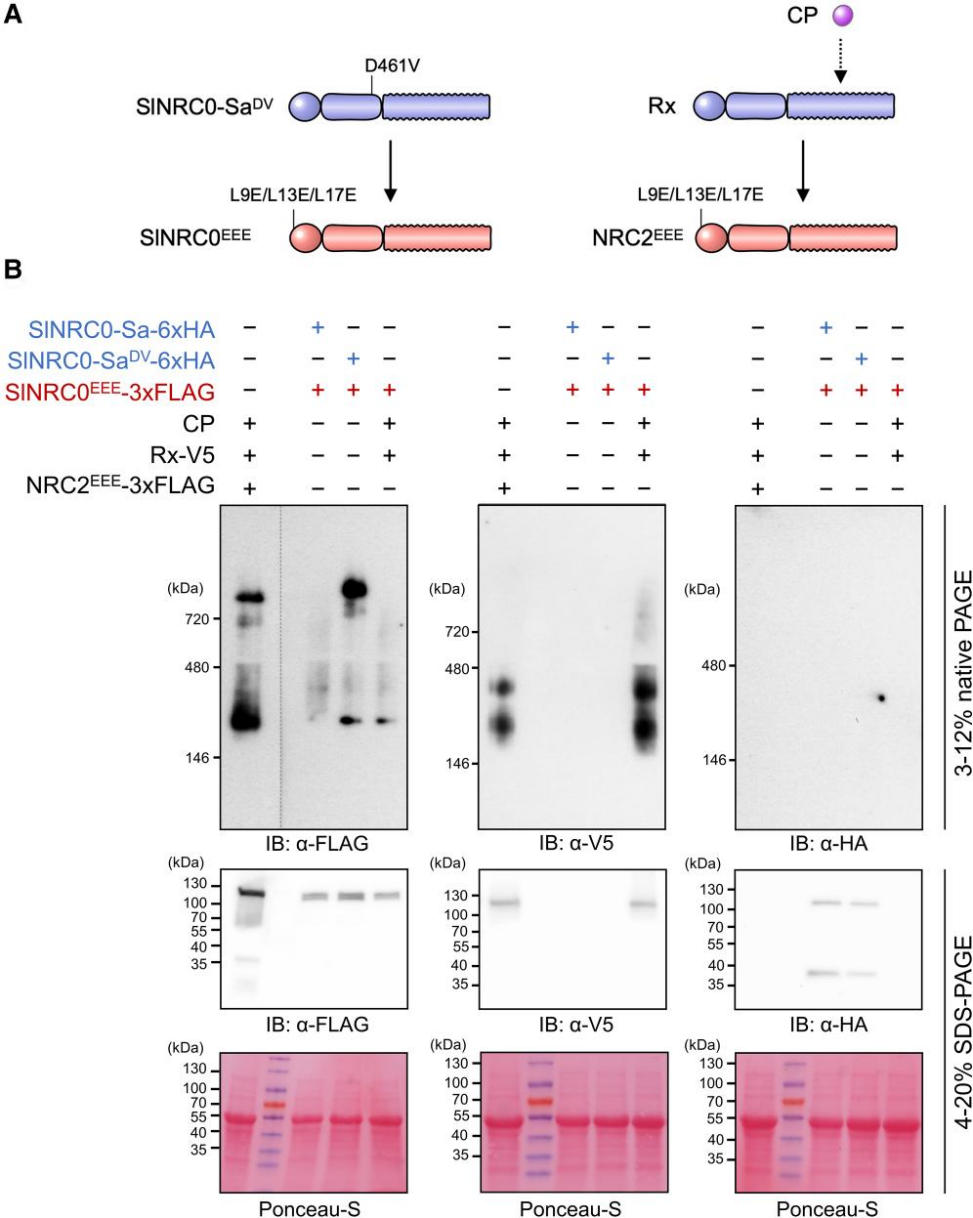
An autoactive NRC0-dependent sensor leads to the formation of an NRC0 higher-order complex in *N. benthamiana*. **A)** A schematic representation of helper NRC activation by sensor NLRs. **B)** The detection of an activated NRC0 complex in BN-PAGE. Each *Agrobacterium* strain carrying a wild-type SlNRC0 sensor (SlNRC0-Sa), a SlNRC0-Sa MHD mutant (SlNRC0-Sa^DV^), a MADA motif mutant of SlNRC0 (SlNRC0^EEE^), *Potato virus X* CP, a wild-type Rx (Rx), or a MADA motif mutant of NRC2 (NRC2^EEE^) was inoculated to the leaves of an *N. benthamiana nrc2 nrc3 nrc4* mutant line. Total proteins were extracted from the inoculated leaves at 3 d after agroinfiltration. Extracts were run on native and SDS–PAGE gels and immunoblotted with anti-FLAG, anti-V5, and anti-HA antibodies, respectively. Loading control was visualized with Ponceau-S staining. The higher-order complex of activated NRC0 was detected in 3 independent experiments.

Given that the activation of NRC-dependent sensors induces homo-oligomerization of helper NRCs in the genetically dispersed NRC network (Ahn et al. 2023; Contreras et al. 2023a), we hypothesized that the activation of NRC0-S leads to oligomerization of its genetic partner NRC0. To test this hypothesis, we first generated a MADA motif mutant of tomato NRC0 (SlNRC0^L9E/L13E/L17E^, referred to as SlNRC0^EEE^; Fig. 8A). This mutation suppresses cell death induction by MADA-type NLRs without inhibiting their resistosome formation (Adachi et al. 2019a; Hu et al. 2020; Förderer et al. 2022; Ahn et al. 2023; Contreras et al. 2023a). We expressed SlNRC0^EEE^ in *nrc2 nrc3 nrc4* knockout *N. benthamiana* lines with wild-type SlNRC0-Sa or its autoactive mutant SlNRC0-Sa^DV^ (Fig. 8A). In an inactive state with wild-type SlNRC0-Sa, SlNRC0^EEE^ was detected as a smear migrating mostly below ∼480 kDa in the blue native polyacrylamide gel electrophoresis (BN-PAGE) assay (Fig. 8B). Upon activation by coexpressing SlNRC0-Sa^DV^, SlNRC0^EEE^ shifted to a slow-migrating higher-molecular-weight complex visible as a band above the 720 kDa marker (Fig. 8B). This higher-order complex band of SlNRC0^EEE^ was not observed in a sample coexpressing Rx and its cognate ligand *Potato virus X* coat protein (CP), while activated Rx and CP coexpression induced oligomerization of NRC2^EEE^ in the control treatment (Contreras et al. 2023a; Fig. 8B). In this BN-PAGE assay, activated SlNRC0 showed a relatively slow migration than the NRC2 oligomer (Fig. 8B). This result suggests that, like other NRC-Hs, activated NRC0 may form the ZAR1 resistosome-type high-order complex.

Contreras et al. (2023a) and Ahn et al. (2023) showed that NRC-dependent sensor NLRs are not present in the high-order complex of activated NRC, thereby proposing a model that the NRC resistosome is a homo-oligomeric complex. To investigate whether the NRC0-dependent sensor NLR is associated with the activated NRC0 complex, we immunoblotted SlNRC0-Sa in the BN-PAGE assay. Although protein accumulation of wild-type SlNRC0-Sa and SlNRC0-Sa^DV^ was confirmed in an immunoblot of the SDS–PAGE assay, both signals were not detected in the BN-PAGE assay immunoblotted by the anti-hemagglutinin (HA) antibody (Fig. 8B). In the control experiment, activated Rx appeared with 2 bands in the range of 146 to 480 kDa, as reported previously (Contreras et al. 2023a; Fig. 8B). In this study, we could not unambiguously determine whether an activated NRC0-dependent sensor integrates the resistosome complex together with its helper NRC0.

## Discussion

**Figure 9.**
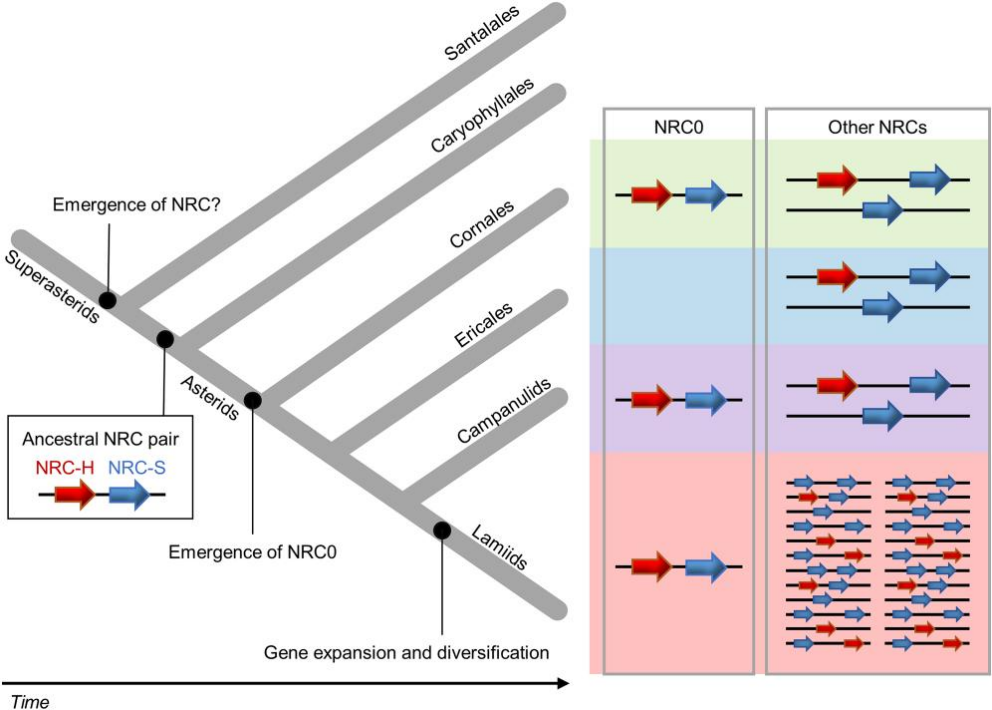
Contrasting patterns of macroevolution in the NRC network of sensor and helper NLRs. The model maps out the key evolutionary transitions in the evolution of NRC-H and NRC-S throughout 125 million years of evolution. The NRC family gene emerged in superasterids possibly before Santalales and Caryophyllales lineages split. The NLR gene cluster of *NRC0* and *NRC0-S* presumably originated from an ancestral NRC gene pair, which emerged before Caryophyllales and asterid lineage split. The *NRC0* gene cluster may have been lost in the Ericales lineage during asterid evolution. The NRC-H and NRC-S genes have expanded and genetically dispersed in lamiid species, while NRC components faced limited expansion in Cornales, Ericales, and campanulids.

NRC-H and NRC-S are phylogenetically related CC-NLRs that form a major superclade in asterid plants that originated from a common ancestor that predates the split between asterids and Caryophyllales (Wu et al. 2017). In solanaceous plants, the monophyletic NRC proteins function as helper NLRs for multiple sensor NLRs in a sister clade and for cell-surface localized immune receptors (Wu et al. 2017; Kourelis et al. 2022; Zhang et al. 2024 ). In this study, we investigated the evolutionary and functional dynamics of NRC0, an atypical member of the NRC family. *NRC0* is the only NRC family member that is conserved across asterid plants with orthologs in 27 species. *NRC0* orthologs are often genetically linked to NRC-S subclade genes. We experimentally validated the functional connections within *NRC0* gene clusters for 4 distantly related asterid species and revealed that *NRC0* is essential for the hypersensitive cell death response triggered by its genetically linked partner *NRC0-S*. Furthermore, activated NRC0-S leads to the formation of an NRC0 high-molecular-weight complex similar to the model reported for other NRC-S/NRC-H pairings. We propose that the *NRC0* sensor/helper gene cluster may reflect an ancestral state that predates the massive expansion of the NRC network in the lamiid lineage of asterid plants. Our findings fill a gap in the evolutionary history of an NLR network in plants and illustrate contrasting patterns of macroevolution within this complex NLR network (Fig. 9).

The *NRC0* gene most likely emerged early in asterid evolution, which corresponds to about 125 mya based on the dating analyses of Wikström et al. (2015) (Fig. 9). A previous phylogenomic study proposed that the NRC superclade expanded from a genetically linked NLR pair over 100 mya before asterids and Caryophyllales lineages split, because an NRC gene cluster exists in sugar beet, a Caryophyllales species (Wu et al. 2017). Consistent with this, we identified 1 NRC-H and 3 NRC-S genes from *B. vulgaris*. However, the sugar beet NRC-H gene did not map to the NRC0 subclade, suggesting that the *NRC0* gene emerged in asterid plants after Caryophyllales and asterid lineages split (Fig. 4B). In this study, we further identified 2 NRC-S genes from a Santalales species, *Malania oleifera*, although we did not find any NRC-H gene in the *Ma. oleifera* reference genome (Fig. 4B) Therefore, the *NRC0* gene cluster probably originated from a common ancestral NLR pair that might be shared with the Caryophyllales NRC gene pair and possibly emerged in the superasterids before Santalales and Caryophyllales lineages split (Fig. 9).

Based on our finding that a Cornales species, *Cor. florida*, has only *NRC0* in the NRC-H subfamily (Fig. 4B), we hypothesize that later during asterid evolution, *NRC0* has duplicated and expanded into complex NRC networks across asterid genomes (Fig. 9). Alternatively, it is still possible that the NRC networks have originated from other ancestral NRC gene pair(s), which have been lost in the asterid lineage. We note that the expansion and diversification of NRC networks are substantial in lamiids. In sharp contrast, Cornales, Ericales, and campanulids have experienced limited expansions of NRC-H and NRC-S genes possibly due to low levels of NRC gene duplications and frequent deletions.

The *NRC0* gene cluster was not found in the Ericales, tea (*Camellia sinensis*), Chinese gooseberry (*Actinidia chinensis* var. chinensis), and *Rhododendron griersonianum* and in some other asterid species such as monkey flower and *N. benthamiana* (Fig. 4B). NLRs are known to be costly genes to plants due to trade-offs between plant growth and NLR-mediated immunity and because they can cause severe autoimmune phenotypes triggered by NLR misregulation (Karasov et al. 2017; Adachi et al. 2019b). Thus, the *NRC0* gene cluster may have been lost as a consequence of selection against potential autoimmunity. Notably, 5 campanulids [lettuce (*Lactuca sativa*), common sunflower (*Helianthus annuus*), bitter vine (*Mikania micrantha*), sweet wormwood (*Artemisia annua*), and horseweed (*Erigeron canadensis*)] and 4 lamiids [European olive (*Olea europaea* subsp. *Europaea*), wild olive (*O. europaea* var. *sylvestris*), trilobed morning glory (*Ipomoea triloba*), and chili pepper (*Capsicum annuum*)] have *NRC0* orthologs but no genetically linked *NRC0-S* encoded within 50 kb genetic distance. It is possible that *NRC0* and *NRC0-S* genes have been genetically dispersed in their genomes like in other sections of the NRC network. Indeed, as recently observed, lettuce NRC0 functions with 3 NRC-S, *Ls124601*, *Ls123301*, and *Ls124100*, whose genes are located on Chromosome 8, 60, 114, and 319 kb away from the *NRC0* gene, respectively (Goh et al. 2023). In addition, NRC0-S is phylogenetically diverse, compared with the well-supported NRC0 subclade (Fig. 3B). This suggests 2 alternative hypotheses: (i) *NRC0-S* genes are evolving faster than *NRC0*, and (ii) sensor NLR genes were repeatedly acquired at the *NRC0* locus and became functionally connected to the NRC0 as NRC0-S. A more precise identification and phylogenomic analyses of *NRC0-S* will provide further evolutionary insights into the *NRC0* sensor–helper gene cluster.

Both NRC0 and NRC0-S across asterids have a typical CC-NB-LRR domain architecture. In the case of well-studied NLR pairs, Arabidopsis RRS1/RPS4, rice RGA5/RGA4, and Pik-1/Pik-2, the sensor NLRs acquired additional integrated domains that function as decoys to bait pathogen effectors (Césari et al. 2014; Le Roux et al. 2015; Maqbool et al. 2015; Sarris et al. 2015; Shimizu et al. 2022; Sugihara et al. 2023). Furthermore, in the Solanaceae NRC networks, about half of the NRC-S subclade members acquired N-terminal domain extensions that are often involved in effector recognition (Saur et al. 2015; Adachi et al. 2019a; Li et al. 2019; Seong et al. 2020). Since NRC0-S does not have additional predicted domains, NRC-S likely recognizes pathogen effectors through its LRR domain as is the case for the ZAR1 and Sr35 CC-NLRs (Wang et al. 2019a, 2019b; Förderer et al. 2022). In terms of effector perception, it is intriguing that the *NRC0* gene cluster is conserved across asterid species over 100 mya. In particular, NLRs are known to exhibit rapid evolution through a birth-and-death model (Michelmore and Meyers 1998). NRC0-S might recognize pathogen effectors in an indirect manner, either monitoring key immune signaling components of the host or functioning with other decoy components.

Our sequence motif analysis revealed that NRC0 orthologs have the MADA motif at their N termini, but their genetically linked NRC0-S partners do not carry the canonical MADA-type sequences of CC-NLRs (Fig. 5). This pattern supports the “use-it-or-lose-it” model in which sensor NLRs lose the molecular signatures of the MADA motif over evolutionary time and instead rely on MADA-type helper NLRs for activation of downstream immune responses (Adachi et al. 2019a). We experimentally demonstrated that NRC0 orthologs can induce the hypersensitive cell death and are required for NRC0-S autoactive cell death (Fig. 6). Although NRC0-S is not predicted to have the MADA motif and does not induce cell death without an NRC0 helper, the “MAHAAVVSLxQKLxx” sequence is conserved at its N termini across NRC0-S proteins (Fig. 5). This conservation pattern is striking because other CC domain sequences have been highly diversified among the NRC0-S (Fig. 5). The N-terminal MAHA-type sequence may have a role in the molecular function of NRC0-S and was, therefore, maintained at its N termini for over 100 million years.

Our BN-PAGE assays revealed that activated NRC0-S induces the formation of NRC0 high-order complexes (Fig. 8). This is consistent with previous findings that activated NRC-S proteins induce the formation of homo-oligomerized NRC2 resistosome (Ahn et al. 2023; Contreras et al. 2023a). In this study, we could not ascertain whether activated NRC0 forms a resistosome-like high-order complex on its own or together with its sensor partner. This was presumably due to protein stability issues with SlNRC0-Sa under the BN-PAGE conditions (Fig. 8). Although further biochemical studies are needed for gaining further mechanistic insights into NRC0 activation, the current results are consistent with the activation-and-release model proposed by Contreras et al. (2023a). In the future, the NRC0 helper–sensor pairs will help map out the evolution of biochemical activation in the NRC network throughout asterid evolution.

In summary, our study helped reveal an ancestral state of the NRC network, resulting in an evolutionary model in which the massively expanded NRC networks evolved from a genetically linked NLR gene pair. As illustrated in Fig. 9, the NRC-type NLRs have experienced contrasting patterns of macroevolutionary dynamics over the last 125 million years from an NLR gene cluster, where the NRC-H is genetically and functionally linked to sensor NLR, to a massive genetically dispersed network. In Solanaceae, the NRC network evolved over tens of millions of years to confer resistance to pathogens and pests as diverse as viruses, bacteria, oomycetes, nematodes, and insects. However, the type of pathogen effectors that are recognized by *NRC0* cluster sensor–helper pairs remains unknown. Furthermore, the structure of paired or networked NLR proteins and the determinants of functional specificities between the sensor–helper NLRs are important unanswered questions in the plant NLR research field. The NRC0 pairs will also help to map out the evolution of activation mechanisms across asterids. Future investigations that contrast the ancestral and modern states of NRC proteins will provide valuable insights into the biochemical function of NLR pairs and networks in plants and how these have evolved over 125 million years.

## Materials and methods

### Phylogenetic analyses

For the phylogenetic analysis, we aligned NLR amino acid sequences using MAFFT v.7 (Katoh and Standley 2013) and deleted the gaps in the alignments by our own Python script. The script is available from GitHub (https://github.com/slt666666/NRC0). NB-ARC domain sequences of the aligned NLR datasets were used for generating phylogenetic trees. The maximum likelihood phylogenetic tree was generated in RAxML version 8.2.12 with Jones-Taylor-Thornton (JTT) model and bootstrap values based on 100 iterations. The best protein substitution model was selected by “­m PROTGAMMAAUTO” option in RAxML. All datasets used for phylogenetic analyses are summarized in Supplementary Files S2 to S12, and raw phylogenetic tree files are summarized in Supplementary Files S13 to S18.

### Patristic distance analyses

To calculate the phylogenetic (patristic) distance, we used Python script based on DendroPy (Sukumaran and Holder 2010). We calculated patristic distances from each NRC to the other NRCs on the phylogenetic tree (Supplementary File S13) and extracted the distances between the NRCs of tomato (*S. lycopersicum*) to the closest NRC from the other plant species. The script used for the patristic distance calculation is available at GitHub (https://github.com/slt666666/NRC0).

### Gene cluster analysis

To calculate the genetic distances, we extracted gene annotation data from the NCBI database as gff3 format. The genetic distances between NLR genes were calculated by our own Python script. The script is available from GitHub (https://github.com/slt666666/NRC0). We defined NLR clusters as NLR sets that genetic distances are <50 kb apart from each other. NLR clusters were visualized in phylogenetic tree by iTOL (Letunic and Bork. 2021). To visualize phylogenetic relationships of clustered NLRs in each species, we developed a “gene-cluster-matrix” library (https://github.com/slt666666/gene-cluster-matrix).

### NRC0 and its sensor candidate sequence retrieval

We performed BLAST (Altschul et al. 1990) using amino acid sequences of NRC0 orthologs from carrot (*D. carota*; DCAR_023561), coffee (*C. canephora*; Cc11_g06560), wild sweet potato (*I. trifida*; itf14g00240.t1 and itf14g00270.t1), and tomato (Solyc10g008220.4.1) as queries to search NRC0-like sequences in NCBI nr or nr/nt database (https://blast.ncbi.nlm.nih.gov/Blast.cgi). In the BLAST search, we used cutoffs, percent identity ≥40%, and query coverage ≥95%. NB-ARC domain sequences of the aligned sequences of the BLAST result were used for generating a phylogenetic tree and extracted NLRs located in the NRC0 clade as NRC0-like sequences. The BLAST pipeline was circulated by using the obtained sequences as new queries to search NRC0-like sequences over the angiosperm species.

We also generated a phylogenetic tree of NLR dataset of each species, in which NRC0-like sequences were found and extracted NLRs located in the NRC0 clade as NRC0-like sequences. Finally, we generated a phylogenetic tree of NRC0-like sequences and NLR dataset of 6 asterid species [Chinese tupelo (*Nyssa sinensis*), tea (*Cam. sinensis*), cardoon (*Cynara cardunculus*), carrot (*D. carota*), sesame (*Sesamum indicum*), and tomato (*S. lycopersicum*)] and defined the NRC0 ortholog clade that includes NRC0 sequences of carrot, coffee, wild sweet potato, and tomato based on phylogenetically well-supported bootstrap value. To extract the *NRC0-S* gene, we extracted genes located within 50 kb from the obtained *NRC0* genes and ran the NLR tracker pipeline (Kourelis et al. 2021) to annotate NLR genes among them.

### Sequence conservation analyses

Full-length amino acid sequences of NRC0 or NRC0-S were subjected to motif searches using MEME (Bailey and Elkan 1994) with parameters “0 or 1 occurrence per sequence, top 20 motifs,” to detect consensus motifs conserved in ≥90% of the input sequences. The output data are summarized in Supplementary Tables S1 and S2.

To analyze amino acid sequence conservation and variation in NRC0 or NRC0-S proteins, aligned amino acid sequences of each NRC0 and NRC0-S datasets by MAFFT v.7 were used for the ConSurf pipeline (Ashkenazy et al. 2016). Tomato NRC0 (XP_004248175.2) or NRC0-Sa (XP_004248174.1) was used as a query for each analysis of NRC0 or NRC0-S, respectively. The output datasets of the ConSurf analyses are included in Supplementary Data Sets 5 and 6.

### Plant materials and growth conditions

Wild-type and mutant *N. benthamiana* were grown in a controlled growth chamber with temperatures ranging from 22 to 25 °C, humidity 45% to 65%, and a 16/8 h light/dark cycle. Fluorescent light bulbs (Slyvania Gro—Lux F58W/Gro—T8 and Phillips master TL-D 58W84D) were used, and the light intensity was about 200 *μ*m/ms^2^. The *NRC* knockout lines used have been previously described: *nrc2 nrc3 nrc4*-210.4.3 and *nrc2 nrc3 nrc4*-210.5.5 (Wu et al. 2020).

### Transient gene expression and cell death assays

Transient gene expression in *N. benthamiana* leaves was performed by agroinfiltration according to methods described previously (Bos et al. 2006). Briefly, 4-wk-old *N. benthamiana* plants were infiltrated with *Agrobacterium* (*Agrobacterium tumefaciens*) Gv3101 strains carrying the binary expression plasmids. The *Agrobacterium* suspensions were prepared in an infiltration buffer (10 mm MES, 10 mm MgCl_2_, and 150 *μ*m acetosyringone, pH 5.6). To overexpress NLRs for cell death assays, the concentration of each suspension was adjusted to OD_600_ = 0.25. Macroscopic cell death phenotypes were scored according to the scale of Adachi et al. (2023). Raw hypersensitive response (HR) index scores can be found in Supplementary Data Set 7.

### Plasmid construction

Wild-type and MHD-mutant variants of *NRC0* and *NRC0-S* from tomato (*S. lycoperiscum*, Sl-), wild sweet potato (*I. trifida*, Itf-), coffee (*C. canephora* or *C. arabica*, Cc- or Ca-), and carrot (*D. carota*, DCAR-) were synthesized through GENEWIZ Standard Gene Synthesis with synonymous mutations to *Bsa*I and *Bpi*I restriction enzyme sites. The synthesized genes were assembled into the binary vector pICH47732 or pICH47751 from the Golden Gate Modular Cloning (MoClo) kit (Weber et al. 2011) together with pICH51266 (35S promoter) and pICH41432 [octopine synthase (OCS) terminator] from the MoClo plant parts kit (Engler et al. 2014).

For BN-PAGE experiment, the MADA motif mutant of *SlNRC0* was amplified by Phusion High-Fidelity DNA Polymerase (Thermo Fisher) with forward mutation primer (AATGGTCTCTAATGGCTGATGCTGTTGTCGAATTTGAATTGTTAAATGAGAAACAACTAGAACTTTATCATGTGGATTTG) and reverse primer (AATGGTCTCTCGAACCAATATCTGGAGGATAGATGAC). The purified amplicon was used for the Golden Gate assembly with pICSL50007 (3xFLAG; Engler et al. 2014) into the binary vector pICH86988 (Level 1 acceptor with 35S promoter and *OCS* terminator; Weber et al. 2011). The synthetic *SlNRC0-Sa^DV^* was also assembled into the pICH86988 binary vector together with pICSL50009 (6xHA) (Engler et al. 2014). The *Rx* and *NRC2^EEE^* constructs used were described previously (Contreras et al. 2023a, 2023b) and were assembled into the pJK268c vector (Kourelis et al. 2020) with C-terminal tag modules pICSL50012 (V5) and pICSL50007 (3xFLAG) (Engler et al. 2014), respectively.

Plasmid information, including constructs generated by previous studies, is described in Supplementary Data Set 8.

### Transcriptome analysis

For the transcriptome analysis of tomato NRCs, raw RNA-seq reads extracted from leaf and root tissues of *S. lycopersicum* published in Lüdke et al. (2023) were used (accession number: SAMN38499990). The obtained RNA-seq reads were filtered and trimmed using fastp (Chen et al. 2018). The quality-trimmed reads were mapped to the reference *S. lycopersicum* genome (SL3.0, ITAG3.2 annotation) using STAR (Dobin et al. 2013). The number of read alignments in the gene regions were counted using featureCounts, and read counts were normalized by using a trimmed mean of *M* values (Liao et al. 2014).

### Protein extraction and BN-PAGE assay

Four-week-old *nrc2 nrc3 nrc4* plants were infiltrated with *Agrobacterium* suspensions, and the concentration of each suspension is indicated in Supplementary Data Set 8. Leaf tissue was collected at 72 h after agroinfiltration in liquid nitrogen and was grounded in a Geno/Grinder homogenizer. Total proteins were extracted in the GTMN buffer [10% v/v glycerol, 50 mm tris-HCl (pH 7.5), 5 mm MgCl_2_, and 50 mm NaCl], supplemented with 10 mm DTT, 1× protease inhibitor cocktail (Sigma-Aldrich), and 0.2% v/v Triton X-100 (Sigma-Aldrich), and incubated on ice for 10 min. After centrifugation at 5,000 × *g* for 15 min, supernatants were used for the BN-PAGE and SDS–PAGE assays.

For BN-PAGE, 25 *µ*L of extracted protein samples were mixed with NativePAGE 5% v/v G-250 sample additive (Invitrogen). Five microliters of each sample were loaded to NativePAGE 4% to 16% w/v bis-tris gels (Invitrogen). Proteins were transferred to PVDF membranes with the NuPAGE Transfer Buffer (Invitrogen) in a Trans-Blot Turbo transfer apparatus (Bio-Rad) by following the manufacturer's instructions. The proteins were fixed to the membranes by incubating with 8% v/v acetic acid for 15 min and were left to dry. For SDS–PAGE, the extracted proteins were diluted in an SDS loading dye and incubated at 72 °C for 10 min and were loaded to 4% to 20% w/v Mini-PROTEAN TGX gels (Bio-Rad). Immunoblotting was performed using the antibodies, anti-HA (3F10) HRP (Roche), anti-V5 (V2260) HRP (Roche), and anti-FLAG (M2) (Sigma), in a 1:5,000 dilution ratio.

### Accession numbers

Genome and gene information used in this study can be found in reference genomes or GenBank/EMBL databases with accession numbers listed in Supplementary Data Sets 1, 4, and 9.

## Supplementary Material

koae154_Supplementary_Data

## Data Availability

All large-scale data are provided in the manuscript and supplementary datasets.

## Supplementary data

The following materials are available in the online version of this article.

**Supplementary Figure S1.** A phylogenetic tree of an NRC-H clade of 6 asterid species.

**Supplementary Figure S2.** Clustered NLRs of 4 asterid species.

**Supplementary Figure S3.** A schematic representation of *NRC0* and NRC sensor gene loci in a *Cor. florida* scaffold.

**Supplementary Figure S4.** NRC0 does not function downstream of NRC2/NRC3-, NRC4-, and NRC2/NRC3/NRC4-dependent sensor NLRs in hypersensitive cell death.

**Supplementary Figure S5.** The NRC0 sensor requires the genetically linked NRC0 but not other NRC family members to trigger hypersensitive cell death.

**Supplementary Figure S6.** A quantification of the autoactive cell death response triggered by multiple combinations of NRC0 and NRC0-S.

**Supplementary Table S1.** A list of MEME motifs predicted from NRC0.

**Supplementary Table S2.** A list of MEME motifs predicted from NRC0-S.

**Supplementary Data Set 1.** A list of *NRC0* and *NRC0-S*.

**Supplementary Data Set 2.** A list of the NLR gene cluster in 4 asterid species.

**Supplementary Data Set 3.** NLR gene cluster matrix files of 4 asterid species used for Supplementary Fig. S2.

**Supplementary Data Set 4.** A list of NRC in 32 asterids, 1 Caryophyllales, and 1 Santalales species.

**Supplementary Data Set 5.** The ConSurf conservation score among NRC0 proteins.

**Supplementary Data Set 6.** The ConSurf conservation score among NRC0-S proteins.

**Supplementary Data Set 7.** A summary of HR index scores in cell death assays.

**Supplementary Data Set 8.** A list of plasmids used in this study.

**Supplementary Data Set 9.** Reference genome databases used for NLR annotations.

**Supplementary File S1.** Amino acid sequences of NLRs in 6 asterid species and functionally validated NLRs used for Fig. 1A.

**Supplementary File S2.** An amino acid alignment file of the NB-ARC domain of NLRs in 6 asterid species and functionally validated NLRs used for Fig. 1A.

**Supplementary File S3.** Amino acid sequences of NLRs in 4 asterid species and functionally validated NLRs used for Fig. 2A.

**Supplementary File S4.** An amino acid alignment file of the NB-ARC domain of NLRs in 4 asterid species and functionally validated NLRs used for Fig. 2A.

**Supplementary File S5.** Amino acid sequences of CC-NLRs in 6 asterid species, NRC0, and NRC0-S used for Fig. 3B.

**Supplementary File S6.** An amino acid alignment file of the NB-ARC domain of CC-NLRs in 6 asterid species, NRC0, and NRC0-S used for Fig. 3B.

**Supplementary File S7.** Amino acid sequences of NRC-Hs used for Fig. 4A.

**Supplementary File S8.** An amino acid alignment file of full-length NRC-Hs used for Fig. 4A.

**Supplementary File S9.** Amino acid sequences of NRC0.

**Supplementary File S10.** Amino acid sequences of NRC0-S.

**Supplementary File S11.** An amino acid alignment file of NB-ARC domains and phylogenetic tree file of each of 32 asterids, 1 Caryophyllales, and 1 Santalales species used for Fig. 4B.

**Supplementary File S12.** Amino acid sequences of NRCs in 32 asterids, 1 Caryophyllales, and 1 Santalales species.

**Supplementary File S13.** An NLR phylogenetic tree file in Fig. 1A.

**Supplementary File S14.** An NLR phylogenetic tree file in Supplementary Fig. S1.

**Supplementary File S15.** An NLR phylogenetic tree file in Fig. 2A.

**Supplementary File S16.** An NLR phylogenetic tree file in Supplementary Fig. S2.

**Supplementary File S17.** An NLR phylogenetic tree file in Fig. 3B.

**Supplementary File S18.** An NLR phylogenetic tree file in Fig. 4A.

## Dive Curated Terms

The following phenotypic, genotypic, and functional terms are of significance to the work described in this paper:

ADR1 Gramene: AT1G33560
ADR1 Araport: AT1G33560

NPR1 Gramene: AT1G64280
NPR1 Araport: AT1G64280

SNC1 Gramene: AT4G16890
SNC1 Araport: AT4G16890

NRG1 Gramene: Niben101Scf02118g00018.1
NRG1 Araport: Niben101Scf02118g00018.1
