# Supplementary material for: The NRC0 gene cluster of sensor and helper NLR immune receptors is functionally conserved across asterid plants
Source: Plant Cell. 2024 Jun 4;36(9):3344–61. doi: 10.1093/plcell/koae154 (PMC11371149; doi:10.1093/plcell/koae154)
Supplement: koae154_Supplementary_Data [file koae154_supplementary_data.zip › Supplementary Data.pdf]

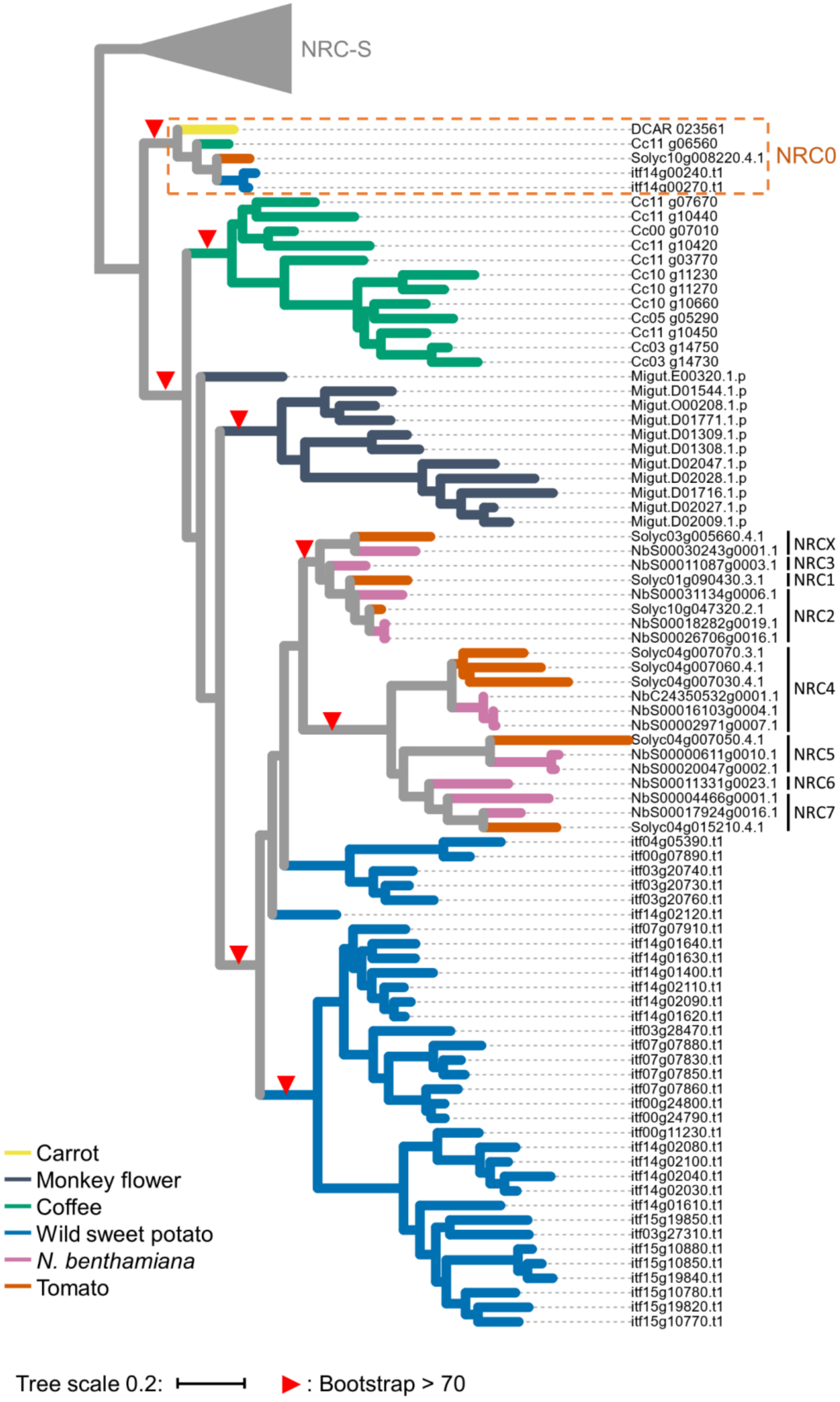

Supplementary Figure S1. Phylogenetic tree of NRC-H clade of six asterid species. (Supports Figure 1.) The phylogenetic rooted tree of NRC-H clade was extracted with the NRC-S clade (an outgroup) from the CC-NLR unrooted tree shown in Figure 1. This tree includes 83 NRC-Hs identified from carrot, monkey flower, coffee, wild sweet potato, *Nicotiana benthamiana*, and tomato. Red arrow heads indicate bootstrap support > 0.7. The scale bars indicate the evolutionary distance in amino acid substitution per site. Each branch is described with different color codes based on plant species: carrot (yellow), monkey flower (black), coffee (green), wild sweet potato (blue), *N. benthamiana* (pink), and tomato (vermilion).

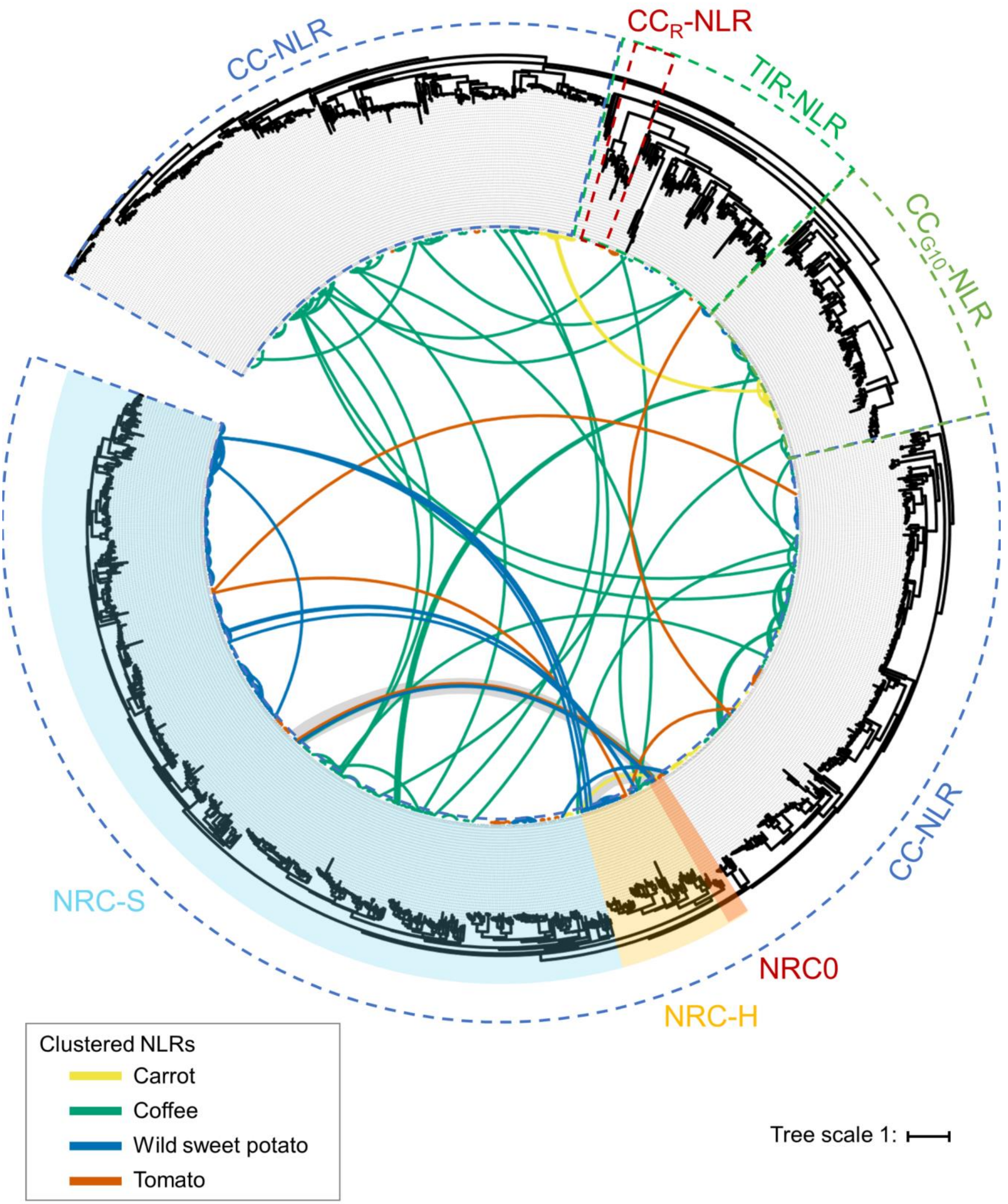

Supplementary Figure S2. Genetically clustered NLRs in four asterid species. (Supports Figure 2.) The phylogenetic unrooted tree was generated in RAxML version 8.2.12 with JTT model using NB-ARC domain sequences of 1,265 NLRs identified from carrot, coffee, wild sweet potato and tomato. The scale bar indicates the evolutionary distance in amino acid substitution per site. The NRC subclades are described with different background colors: NRC0 (red), other NRC-H (orange), and NRC-S (light blue). The connected lines between nodes indicate genetically linked NLRs (distance < 50 kb) with different colors based on plant species: carrot (yellow), coffee (green), wild sweet potato (blue), and tomato (vermilion). Gray band indicate genetic link between *NRC0* and *NRC0-S*.

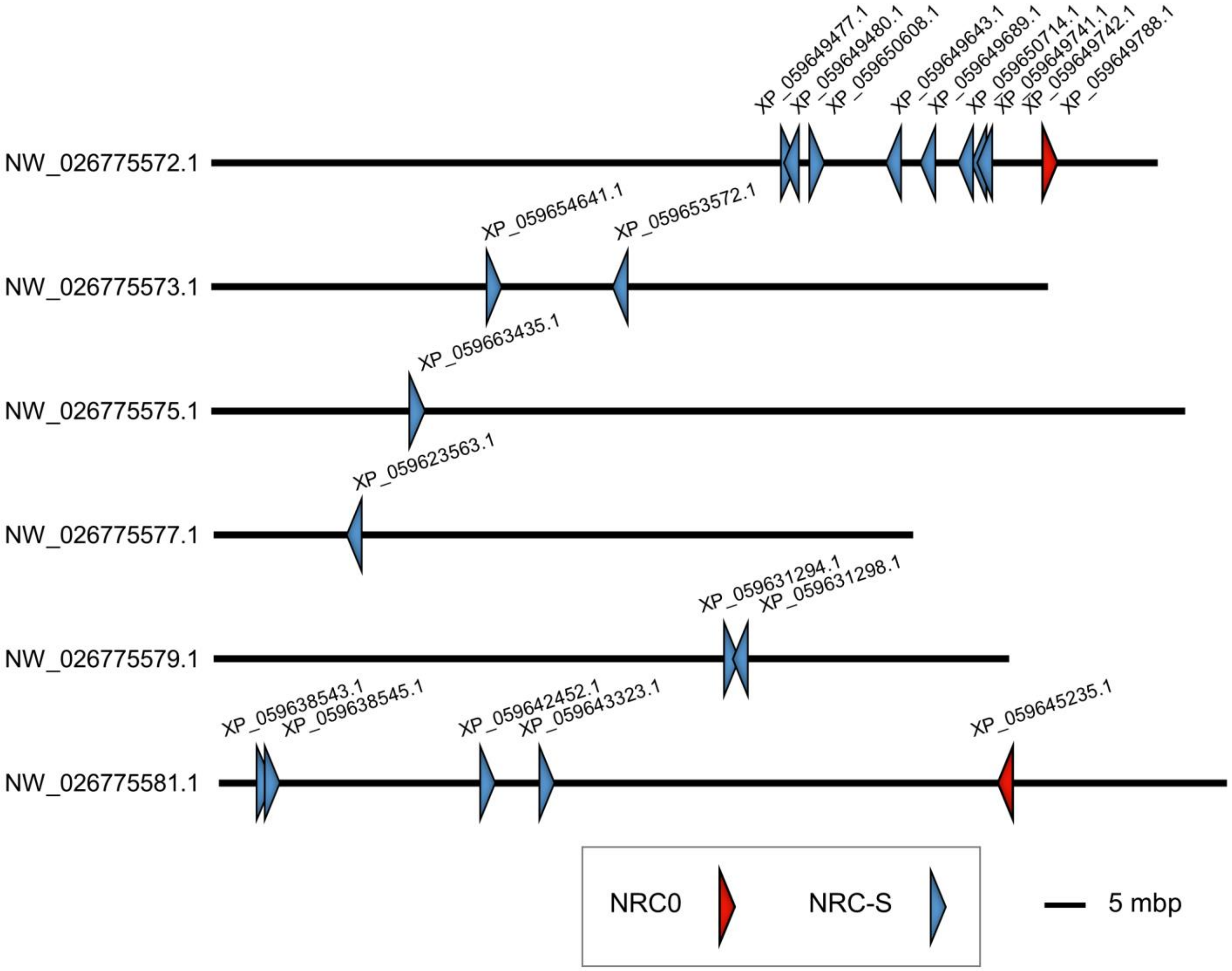

Supplementary Figure S3. Schematic representation of *NRC0* and NRC sensor gene loci in *Cornus florida* scaffold. (Supports Figure 4.) *NRC0* and NRC sensor (NRC-S) genes are highlighted in red and blue, respectively.

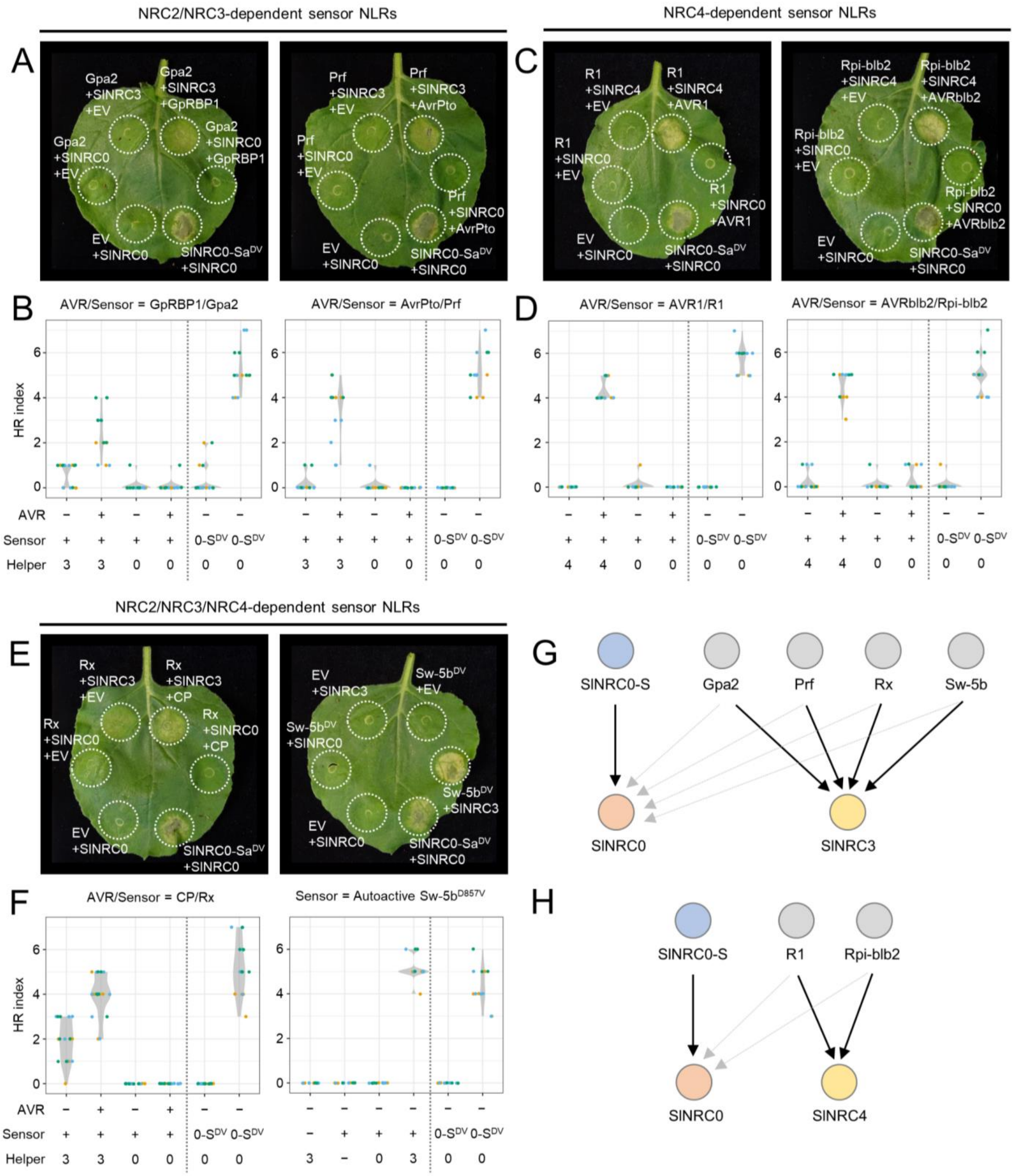

Supplementary Figure S4. NRC0 does not function downstream of NRC2/NRC3-, NRC4- and NRC2/NRC3/NRC4-dependent sensor NLRs in hypersensitive cell death. (Supports Figure 6.) Wild-type SlNRC0 was co-expressed in *nrc2 nrc3 nrc4* knockout *N. benthamiana* leaves with NRC2/NRC3-dependent sensor NLRs **(A)**, NRC4-dependent NLRs **(C)** and NRC2/NRC3/NRC4-dependent sensor NLRs **(E)**, respectively. To activate each sensor NLR (Sensor), cognate avirulence (AVR) genes were co-expressed, or the autoactive Sw-5b mutant (Sw-5b^{D857V}) was expressed by agroinfiltration. Wild-type SlNRC3 and SlNRC4 were used as helper NLR controls (Helper) to SlNRC0, and empty vector (EV) was used as a negative control. Cell death phenotype was recorded five days after the agroinfiltration. **(B, D, F)** Violin plots showing cell death intensity scored as an HR index based on 12 (Sw-5b^{D857V}), 13 (AvrPto/Prf and AVR1/R1), 15 (AVRblb2/Rpi-blb2), 16 (GpRBP1/Gpa2) and

17 (CP/Rx) replicates (different leaves from independent plants) in three independent experiments of A, C and E. Each experiment is visualized with different dot colors. **(G, H)** Schematic representation describing tomato helper NRC dependency of sensor NLRs tested in panels A to F. Black arrows indicate functional connections between sensor and helper NLRs, while gray dot arrows describe functional mismatches of sensor-helper pairings in *N. benthamiana* leaves.

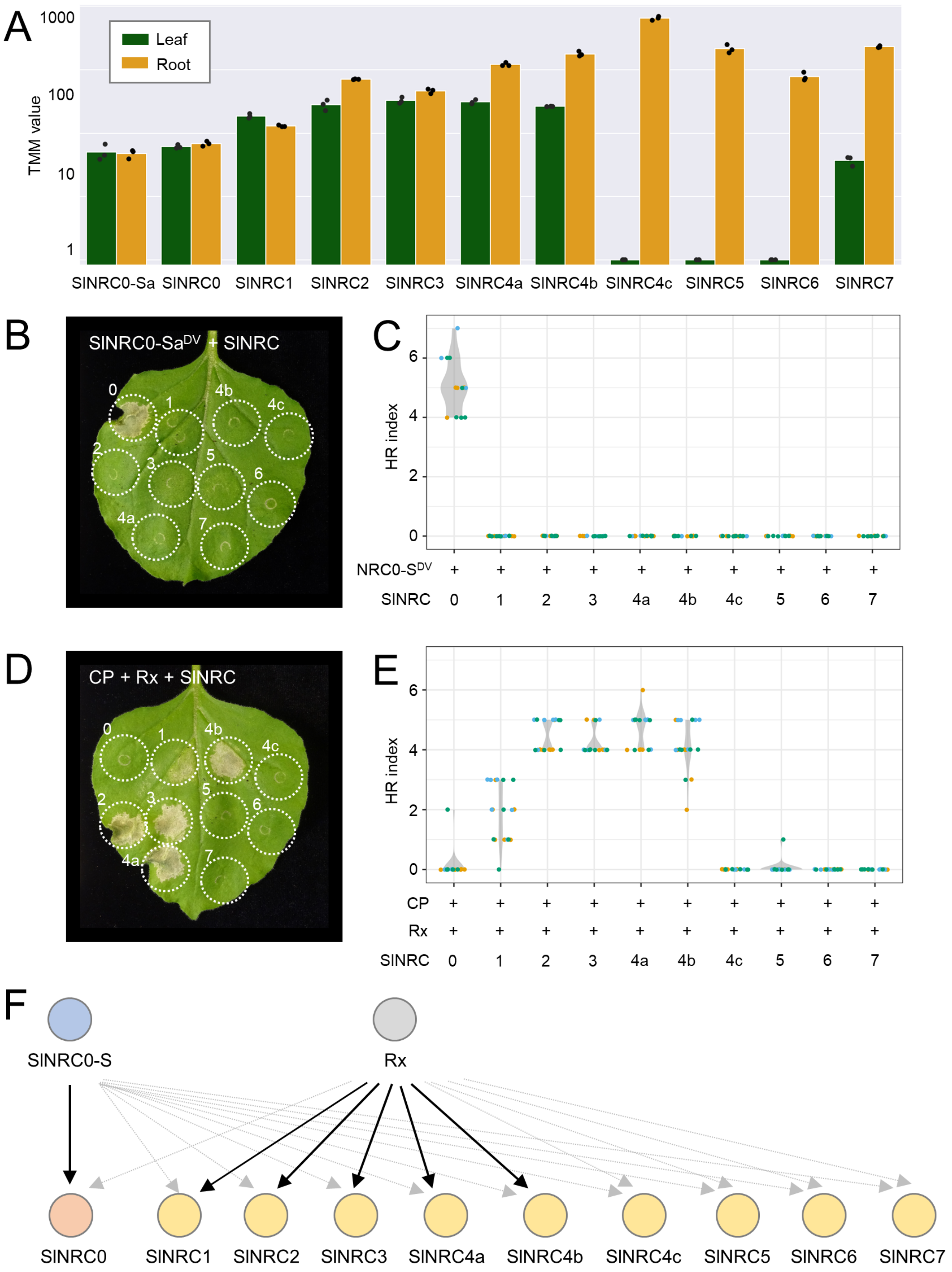

Supplementary Figure S5. NRC0 sensor requires the genetically linked NRC0, but not other NRC family members to trigger hypersensitive cell death. (Supports Figure 6.) **(A)** Transcriptome profile of tomato *NRC0-Sa* and *NRCs* in root and leaf tissues. TMM normalized counts are described for the *SlNRC0-Sa*, *SlNRC0*, *SlNRC1*, *SlNRC2*, *SlNRC3*, *SlNRC4a*, *SlNRC4b*, *SlNRC4c*, *SlNRC5*, *SlNRC6* and *SlNRC7*, respectively. **(B)** Wild-type SlNRC0 and other SlNRC helpers were co-expressed in *nrc2 nrc3 nrc4* knockout *N. benthamiana* leaves with the autoactive MHD mutant of SlNRC0 sensor (SlNRC0-S^{DV}). Cell death phenotype was recorded five days after the agroinfiltration. **(C)** Violin plots showing cell death intensity scored as an HR index based on 15 replicates (different

leaves from independent plants) in three independent experiments of A. Each experiment is visualized with different dot colors. **(D)** As a control experiment to panel A, wild-type SlNRC0 and other SlNRC helpers were co-expressed in *nrc2 nrc3 nrc4* knockout *N. benthamiana* leaves with sensor NLR Rx and *Potato virus X* coat protein (CP). Cell death phenotype was recorded five days after the agroinfiltration. **(E)** Violin plots showing cell death intensity scored as an HR index based on 18 replicates (different leaves from independent plants) in three independent experiments of C. Each experiment is visualized with different dot colors. **(F)** Schematic representation describing tomato helper NRC dependency of the SlNRC0 sensor. Black arrows indicate functional connections between sensor and helper NLRs, while gray dot arrows describe functional mismatches of sensor-helper pairs in *N. benthamiana* leaves.

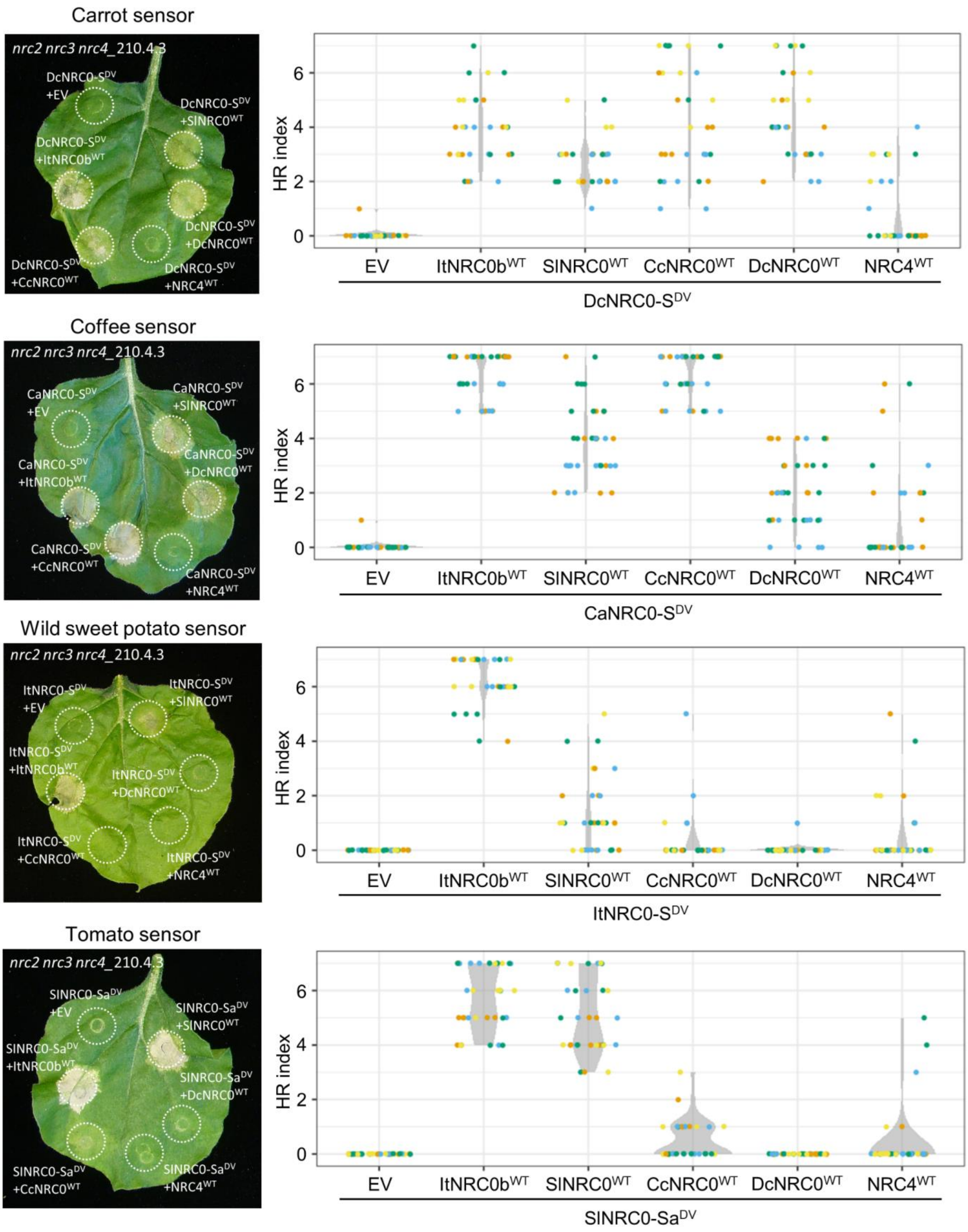

Supplementary Figure S6. Quantification of the autoactive cell death response triggered by multiple combinations of NRC0 and NRC0-S. (Supports Figure 7.) Left images are identical to representative images of autoactive cell death shown in Figure 7. Violin plots describe cell death intensity scored as an HR index based on 16 replicates (different leaves from independent plants) in four independent experiments (carrot, wild sweet potato and tomato sensors) and 18 replicates (different leaves from independent plants) in three independent experiments (coffee sensor). Each experiment is visualized with different dot colors.

Supplementary Table S1. List of MEME motifs predicted from NRC0.

Motif ID	Motif logo	E-value	Start	End	Query ID	Domain	Known motif
Motif_1	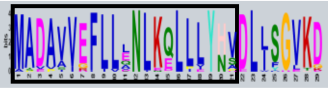	1.2e-819	1	29	XP_004248175.2	CC	MADA
Motif_2	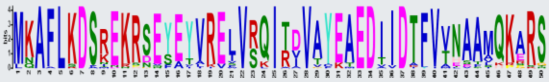	8.5e-1571	41	90	XP_004248175.2	CC	
Motif_3	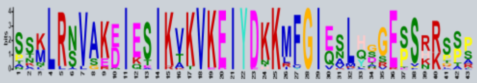	2.1e-1009	102	144	XP_004248175.2	CC	
Motif_4	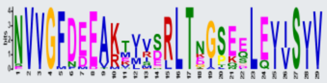	2.0e-743	155	183	XP_004248175.2	NB-ARC	
Motif_5	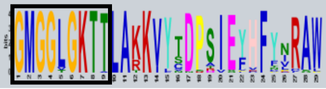	1.4e-1035	184	212	XP_004248175.2	NB-ARC	P-loop
Motif_6	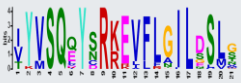	8.4e-511	213	233	XP_004248175.2	NB-ARC	
Motif_7	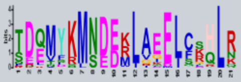	1.3e-451	236	256	XP_004248175.2	NB-ARC	
Motif_8	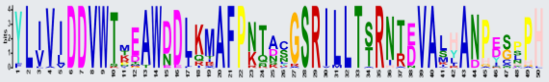	3.6e-1620	260	309	XP_004248175.2	NB-ARC	
Motif_9	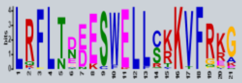	1.2e-538	311	331	XP_004248175.2	NB-ARC	
Motif_10	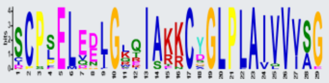	1.0e-806	332	360	XP_004248175.2	NB-ARC	
Motif_11	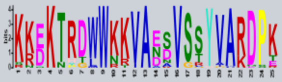	1.1e-734	364	388	XP_004248175.2	NB-ARC	
Motif_12	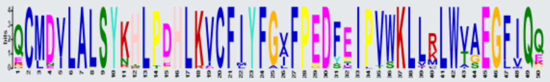	1.0e-1829	389	438	XP_004248175.2	NB-ARC	
Motif_13	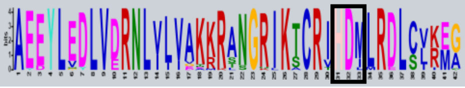	1.2e-1328	448	489	XP_004248175.2	NB-ARC	MHD
Motif_14	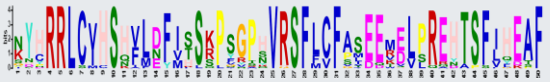	2.5e-1427	512	561	XP_004248175.2	LRR	
Motif_15	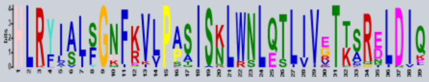	1.8e-1091	586	624	XP_004248175.2	LRR	
Motif_16	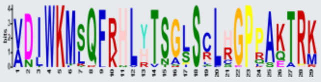	2.7e-791	625	653	XP_004248175.2	LRR	
Motif_17	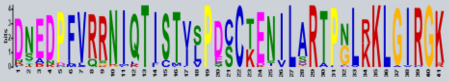	3.4e-1297	654	694	XP_004248175.2	LRR	
Motif_18	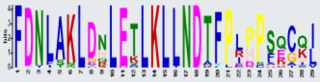	7.3e-782	708	736	XP_004248175.2	LRR	
Motif_19	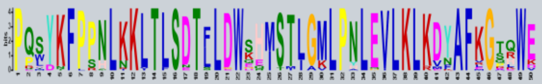	1.2e-1677	740	789	XP_004248175.2	LRR	
Motif_20	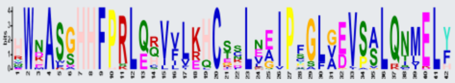	1.6e-1140	810	851	XP_004248175.2	LRR	

Supplementary Table S2. List of MEME motifs predicted from NRC0-S.

Motif ID	Motif logo	E-value	Start	End	Query ID	Domain	Known motif
Motif_1	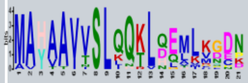	5.6e-189	1	21	XP_004248174.1	CC	
Motif_2	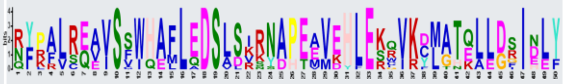	1.1e-385	23	72	XP_004248174.1	CC	
Motif_3	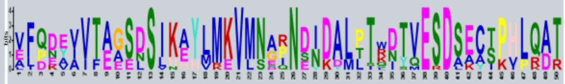	9.1e-401	88	137	XP_004248174.1	CC	
Motif_4	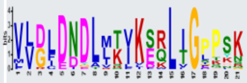	1.1e-196	138	158	XP_004248174.1	NB-ARC	
Motif_5	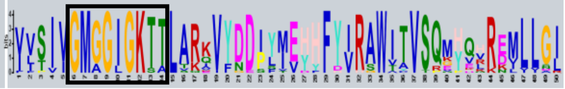	2.8e-842	161	210	XP_004248174.1	NB-ARC	P-loop
Motif_6	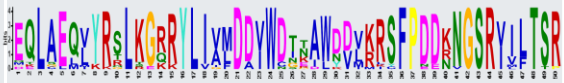	2.0e-749	227	276	XP_004248174.1	NB-ARC	
Motif_7	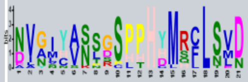	1.2e-124	279	299	XP_004248174.1	NB-ARC	
Motif_8	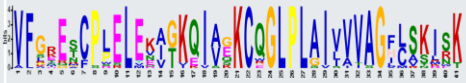	3.3e-451	309	349	XP_004248174.1	NB-ARC	
Motif_9	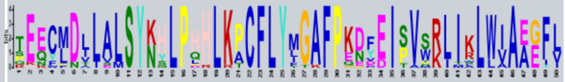	6.4e-627	369	418	XP_004248174.1	NB-ARC	
Motif_10	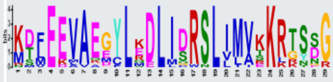	2.4e-276	424	452	XP_004248174.1	NB-ARC	
Motif_11	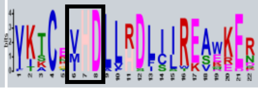	1.8e-203	454	475	XP_004248174.1	NB-ARC	MHD
Motif_12	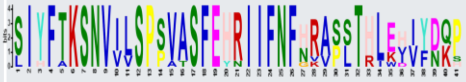	5.6e-214	476	516	XP_004248174.1	LRR	
Motif_13	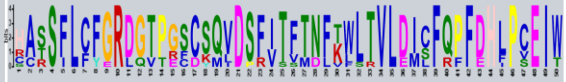	1.7e-348	520	569	XP_004248174.1	LRR	
Motif_14	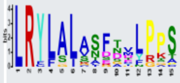	7.9e-84	574	588	XP_004248174.1	LRR	
Motif_15	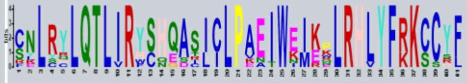	2.5e-222	590	630	XP_004248174.1	LRR	
Motif_16	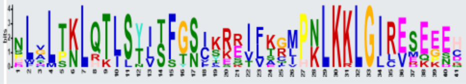	2.5e-224	668	708	XP_004248174.1	LRR	
Motif_17	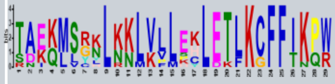	4.8e-113	710	738	XP_004248174.1	LRR	
Motif_18	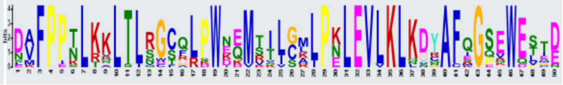	1.0e-429	744	793	XP_004248174.1	LRR	
Motif_19	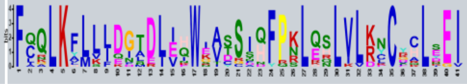	8.3e-275	796	836	XP_004248174.1	LRR	
Motif_20	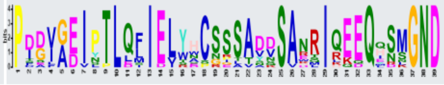	6.9e-295	837	875	XP_004248174.1	LRR	
