# Supplementary material for: The NRC0 gene cluster of sensor and helper NLR immune receptors is functionally conserved across asterid plants
Source: Plant Cell. 2024 Jun 4;36(9):3344–61. doi: 10.1093/plcell/koae154 (PMC11371149; doi:10.1093/plcell/koae154)
Supplement: koae154_Supplementary_Data [file tpc.01082.2023-s31.pdf › tpc.01082.2023-s31.pdf]

THE PLANT CELL

The NRC0 gene cluster of sensor and helper NLR immune receptors is functionally conserved across asterid plants

Toshiyuki Sakai, Mauricio Contreras, Claudia Martinez-Anaya, Daniel Lüdke, Sophien Kamoun, Chih-Hang Wu, and Hiroaki Adachi

Review Timeline:

Submission Date:	13-Nov-2023
Editorial Decision:	10-Jan-2024
Revision Received:	11-Mar-2024
Editorial Decision:	01-Apr-2024
Revision Received:	29-Apr-2024
Accepted:	01-May-2024

Dr. Hiroaki Adachi
Kyoto Daigaku
Muko
Japan

Dear Dr. Adachi:

We have received reviews of your manuscript entitled "The NRC0 gene cluster of sensor and helper NLR immune receptors is functionally conserved across asterid plants." Thank you for submitting your best work to The Plant Cell. The editorial board agrees that the work you describe is substantive, falls within the scope of the journal, and may become acceptable for publication, pending revision and potential re-review.

We ask you to pay particular attention to the two following points in preparing your revision:

The phylogenetic and genomic analyses of NRC0 should be strengthened and the alternative hypotheses regarding the evolution (and origin) of this cluster more explicitely discussed (see comments from reviewer 2 and 3).

The ability of NRC0 to facilitate the action of other (non-clustered) sensor NLRs should be explored.

Please contact us if there are ambiguous comments or if you wish to discuss the revision.

Given the nature of the comments, we are offering you 60 days from when we have issued this decision to complete the revision. If a revision is not returned within this time frame, and if you have not been granted an extension, we will withdraw the manuscript, which will leave you free to submit the work elsewhere. If you need an extension, we encourage you to contact us at any point before the 60 days have passed.

When you are ready to submit the revised version, please upload a highlighted copy that indicates all changes made in response to the editor and reviewer recommendations. Include an itemized list of all changes made in response to each of the reviewer's suggestions in the "Response to Reviewers" section; please note that reviewers do not have access to your cover letter, nor was this decision letter shared with them.

Thank you for the privilege of reviewing your work. We look forward to receiving your revised manuscript.

Sincerely,

The Plant Cell Editorial Board

(Please note that due to a potential conflict of interest, EIC Blake Meyers did not participate in evaluating this manuscript).

Please note the following:

-The Plant Cell now requires authors to complete and submit an author revisions checklist upon submission of a revised manuscript. The aim of the checklist is to aid authors in preparing a high-quality manuscript, facilitate the review and assessment of revised manuscripts, and help to ensure that journal standards are maintained across the board. If your manuscript is accepted, the completed checklist will be published as supplemental material attached to the article online. Please download a copy of the checklist (pdf fillable form) at this link, for submission with your revised manuscript: https://tpc.msubmit.net/html/Author_Revisions_Checklist.pdf.

-Supplemental materials should be restricted to large datasets and tables, presentation of replicates, and validation of reagents, methods, or genotypes. Any data that are used to support the major claims must be in the main manuscript. Supplemental figure legends must indicate what figure in the main manuscript is supported by the supplemental data presented. Please justify how each of the supplemental figures meet the criteria.

-Sampling methods and nature of "biological replicates" should be described precisely (i.e. different plants, parts of plants, pooled tissue, independent pools of tissue, sampled at different times, etc), along with a clear description of and rationale for any statistical analyses conducted. The reader should know exactly what was sampled; what forms the basis of the calculation of any means and statistical parameters reported. This is also necessary to ensure that proper statistical analysis was conducted.

-Want to add this revision deadline to your calendar? Click below!

--- Reviewer comments:

Reviewer #1 (Comments for the Author):

Review for TPC-LBS-01082 NRC0 cluster
In this manuscript the authors dived deeper into the evolution of the NRC-helper sensor network with a focus on one NRC helper member - NRC0- that the authors found to be conserved across asterid plants. The authors discovered also that the NRC0 is genetically linked with NRC(s) that belong o the NRC-sensor (NCR0-S) family. There phyllogentic analysis suggest that the NRC0/NRC0-S gene pair originated from a ancestral gene pair that also gave rise to the highly expanded NRC-H NRC-S network found in lamiids. However, the NRC0 NRC0-S gene pair did not diversify so much and still exist as such gene pair or gene cluster in other asterids. The authors did also perform some cool mechanistic analysis to demonstrate that the NRC0 NRC-S pairs/clusters function together where the NRC-S activates the NRC0 to initiate a cell death response. The authors also show that the 'more ancestral' NRC0s can initiate cell death by being activated by also more distantly related NRC0-S but that the 'younger' NRC0s are unable to be activated by more distantly related NRC0-Ss.
The phylogenetic analysis seem to be accurately performed and are very well documented (methods section). The interpretations are easily formulated and easy to comprehend. I very much like the mechanistic analysis to show the dependency of the NRC0-S partners of the NRC0s in the second part of the manuscript. This analyses nicely confirm their evolutionary findings and - as the authors also write in the discussion - opens up many possibilities to study how NRC-Ss may activate NRC-Hs.
I do have some minor issues/suggestions:
The authors write that some species (including N. benthamiana) have lost the NRC0 gene cluster and that this may be due to autoimmunity or cost trade-offs. Could it be possible that in the species where only NRC0s are found (without the linked NRC0-S genes that these NRC0s have no canonical MADA motif? Probably the authors know or have this information. If these NRC0s have a canonical MADA motif t would be great to see whether these are active (make the DV mutant and test for cell death in N. benthamiana). If these NRC0s are nonfunctional this result could confirm the hypothesis that they are costly to have and thus only inactive versions or no NRC0 is kept.
Is there any support for transcriptional co-regulation of the NRC0 and NRC0-S NLRs? They are clustered but not liked in a head-to-head orientation like the RPS4/RRS1 type of pairs. Would be interesting to have/see some data on this if available. This would also further support their functional relationship.
BN-PAGE experiment: Could it be possible that the aHA antibody is not working for BN-PAGEs? Did the authors try with a different tag or with swapped tags?

Reviewer #2 (Comments for the Author):

Plant immune receptors often exist as diverse series of alleles within a species, but their evolution at the genus, family, and superfamily scales is only beginning to be explored. The manuscript advances this goal by exploring the evolution of a specific helper NLR, describing early diverging orthologs of the NRC clade of NLR receptors. Extensive previous work on the NRC clade in Solanaceae has shown that helper NRCs (NRC-H) are genetically required for responses downstream of many sequence-related sensor NLRs termed "NRC-S". Here the authors show that the most early-diverging NRC-H, termed NRC0, has functional orthologs in outgroup Asterid species. NRC0 also frequently falls in a genetic cluster with sensor NLRs, and the clustered NLR pairs function similarly to the dispersed sensor-helper pairs described previously: autoactivating mutations in the sensors require the helper as shown using the N.benthamiana cell death assay and BN-PAGE analysis of helper NLR molecular complexes. Functional compatibility of NRC0 and NRC0-S orthologs is explored, but not extended combinations of NRC sensors and helpers (see below).

Overall, the evolutionary analyses are excellent and presented very clearly. I especially like the visual representation of cluster evolution with a circos plot in Fig 2A, which nicely shows both lineage-specific and more ancient, conserved clusters. Importantly the work sets another benchmark for the diversification rate of an ancient helper NLR including in specific motifs / subdomains. Alongside the separate case study of ZAR1 this could help provide predictive indicators for helper NLR status.

An ancient, stable sensor-helper pair, genetically sister to a diversified network, would be very interesting. Strong claims are made that the gene cluster is ancient, not just NRC0 itself (lines 39, 153, 332, 406). Unfortunately the claim regarding NRC0 cluster as an "ancestral state that predates [expansion of NRC network in lamiids]" is not well supported. Several lines of evidence refute the "ancestral state" hypothesis:

1. The hypothesis would predict that outgroup species would encode NRC0 homologs but not other NRC genes. In fact,

the exact opposite case is true -- beet, tea, kiwi, and rhododendron encode other NRC-H/S genes, but not NRC0 (Fig 4, Fig 9).

2. The hypothesis would predict that not only NRC0 but also the paired sensors (NRC0-S) would also be related by orthology, thus NRC0-S would form an ancient, outgroup clade sister to NRC-S genes. Instead, phylogenetically distant NRC0-S genes exist at the NRC0 locus (Fig 3), suggesting repeated acquisition of diverse sensors at the locus. While still interesting, this suggests that NRC0 is just acting like the other well-characterized NRCs, it just happens to be the oldest MADA-containing helper. Is there anything special about the NRC0-S genes which happen to function via NRC0?

3. The NRC0 locus is not always a cluster; it is a single gene without NRC0-S homologs in the outgroup lamiids and campanuiids. Since the clustered sensor NLR is not stable (point 2 above), the ancestral state was probably a locus encoding only NRC0, not a cluster.

Overall, I agree that NRC0 is an ancient, slowly evolving NRC-H. While still quite interesting, its function should be shown in the context of the broader NRC network. The critical missing experiments are A) whether NRC0 can enable function of NRC-S genes besides those encoded at the locus itself (such as Rx Bs2 or Prf), and B) whether autoactive NRC0-S function can be enabled by other NRCs. (NRC4 is tested in Fig 7 but not other genes.)

If NRC0 facilitates the activity of non-clustered sensor NLRs, not just NRC0-S genes, the major claim of the manuscript should be revised: NRC0 would be an example of a helper with a stable function and no evidence of diversification, but it would still function within a diverse network, not function as a separate sub-cluster.

Other comments:
- Synteny analysis should be performed in order to confirm loss of NRC0 locus in the Ericales. Genes bordering NRC0 are expected to show synteny with Ericales and related asterids, but with a synteny gap at the NRC0 locus in the Ericales. Without this positive evidence for an NRC0 deletion it is difficult to conclude the absence of NRC0 solely based on BLAST analysis.
- inset panel in Fig 1A and Suppl Fig S1: it is important to show where the NRC clade is rooted to show that NRC0 are the outgroup of all other NRCs. Could the supplemental fig show the root, or an even further outgroup? The main figure legend should refer to the suppl. fig for rooting.
- Fig 9: The NRC0 loci are not identical, as indicated in the figure. The lamiids subpanel is also confusing to me. Do the different lines indicate different lamiid species? Or does the panel indicate a single species with 24 NRC loci?

Reviewer #3 (Comments for the Author):

The manuscript by Sakai et al. reports on the identification of a NRC gene pair including a helper immune receptor termed NRC0 and a cognate sensor NRC0-S that are broadly conserved across asterid plants. The ancestral NRC0 - NRC0-S pair is proposed to be the ancestral state of diversified helper-sensor modules/network found in modern asterids because this pair is the most conserved identified in asterid genomes and several modern NRC0 - NRC0-S pairs show similar signaling ability.

Research on plant immune receptor is a very active field. The architecture and evolution of immune receptor netwoks adressed in this study is timely and relevant. The analyses and experiment are carefully conducted and reported clearly. The methods and discussion sections should be completed/revised and some claims and conclusions should be nuanced as detailled hereafter.

Major comments:

1. Genome mining and phylogenetic analyses identified NRC0 as the only NRC gene conserved in 4 out of 6 asterid species. Some details would be useful to provide in the M&M for this section:
- how was the substitution model selected?
- how many informative sites were selected from the alignments?
- are the trees rooted, if yes which sequence was used as outgroup?

2. Experiments in Nicotiana benthamiana indicate that NRC0 - NRC0-S pairs form functional signaling modules conserved in serveral asterids. NRC0 from carrot and coffe signal with NRC0-S from carrot and coffee but not orthologs from sweet wild potato and tomato, suggesting some degree of co-evolution. If NRC0 was the closest descendent of the ancestral NRC network, I would expect the carrot and coffee alleles, from species that diverged earlier, to show a lower degree of specialization regarding the NRC0 sensors they can signal with. What does the observed specialization pattern tell us about the proposed ancestral NRC network?

3. Throughout the manuscript, some claims regarding the NRC network evolution extrapolate on the actual evidence.

They should be further discussed and nuanced (or additional data provided if available):

"functionally conserved NRC0 gene cluster" > the signaling function downstream of NRC0-S is indeed conserved as shown in this work. However it is not known whether the recognition range of the NRC0 - NRC0-S module is conserved, as mentioned in the manuscript's discussion.

"the NRC0 sensor-helper gene cluster reflects the ancestral state of the NRC network":
- NRC gene deletion might be frequent, as observed for NRC0 in Ericales, so that the Solanales/asterid ancestor species might have included a more complex network than can be inferred based on conserved genes alone.
- Whether other (clustered) NRCs derive from the NRC0 - NRC0-S pair remain to be established. The phylogenetic tree from figure 4A seem to suggest that since the split with Cornales, NRC0 and other NRC-H already formed two distinct clades.
- Can NRC0 signal (trigger cell death) with sensors outside the NRC0-S clade? This would provide support for NRC0 function pre-dating the divergence of the NRC0 clade.
Alternative ancestral NRC network architectures might be worth discussing in the light of these questions/information.

Reviewing Editor comments:

Dear Dr. Pierre-Marc Delaux and Dr. Roger Innes,

Thank you very much for your evaluation of our manuscript " The *NRC0* gene cluster of sensor and helper NLR immune receptors is functionally conserved across asterid plants " for consideration as a Large-Scale Biology article at The Plant Cell.

We appreciate the feedback of the independent reviewers. We have endeavoured in this revision to address as much as possible the pertinent points they raised. Please see the point-by-point 'Response to Reviewers' attached to this file.

We hope that we have addressed many of the issues that were raised, and we look forward to your response.

Best regards,

Hiroaki Adachi

We have received reviews of your manuscript entitled "The NRC0 gene cluster of sensor and helper NLR immune receptors is functionally conserved across asterid plants." Thank you for submitting your best work to The Plant Cell. The editorial board agrees that the work you describe is substantive, falls within the scope of the journal, and may become acceptable for publication, pending revision and potential re-review.

We thank the editors and reviewers for their overall positive evaluation.

We ask you to pay particular attention to the two following points in preparing your revision:

The phylogenetic and genomic analyses of NRC0 should be strengthened and the alternative hypotheses regarding the evolution (and origin) of this cluster more explicitely discussed (see comments from reviewer 2 and 3).

Thank you very much for your comment. In this revision, we included a Cornales species, Cornus florida, in the computational analysis. C. florida has only NRC0 (XP_059649788.1 and XP_059645235.1), but not other NRC-H (Figure 4B). Therefore, we propose that the NRC0 is an ancestral helper gene that predates the massive expansion of the NRC network in the lamiid lineage of asterid plants.

In terms of NRC0-S, our computational pipeline to identify NRC0-S is still technically limited, because functional helper/sensor pairs are not always clustered within less than 50 kb genetic distance and our analysis is dependent on quality of reference genome assembly and gene annotation. Therefore, we toned down our conclusion about the evolution of NRC0-S and NRC0/NRC0-S gene cluster throughout the manuscript.

The ability of NRC0 to facilitate the action of other (non-clustered) sensor NLRs should be explored.

Thank you very much for your comment. In this revision, we performed co-expression of SlNRC0 and six representative sensor NLRs, Gpa2, Prf, R1, Rpi-blb2, Rx and Sw-5b, with or without cognate AVRs in side-by-side experiments. We have this new data in Supplemental Figure S4 of the revised manuscript. In the experiments, SlNRC0 did not function with the functionally validated sensor NLRs in cell death response, while SlNRC3 and SlNRC4 complemented cell death response triggered by matching sensor NLRs. Furthermore, we co-expressed the autoactive mutant of SlNRC0-S, genetically linked sensor to SlNRC0, with nine other tomato NRCs (Supplemental Figure S5). Unlike SlNRC0, the tested SlNRC helpers did not function with SlNRC0-S to trigger cell death in *N.*

benthamiana. These new data support our conclusion that NRC0-S require the genetically linked NRC0 to trigger immune response. NRC0 and NRC0-S form a sub-network independent of the previously described NRC network.

---------- Reviewer comments:

Reviewer #1 (Comments for the Author):

In this manuscript the authors dived deeper into the evolution of the NRC-helper sensor network with a focus on one NRC helper member - NRC0- that the authors found to be conserved across asterid plants. The authors discovered also that the NRC0 is genetically linked with NRC(s) that belong o the NRC-sensor (NCR0-S) family. There phyllogentic analysis suggest that the NRC0/NRC0-S gene pair originated from a ancestral gene pair that also gave rise to the highly expanded NRC-H NRC-S network found in lamiids. However, the NRC0 NRC0-S gene pair did not diversify so much and still exist as such gene pair or gene cluster in other asterids. The authors did also perform some cool mechanistic analysis to demonstrate that the NRC0 NRC-S pairs/clusters function together where the NRC-S activates the NRC0 to initiate a cell death response. The authors also show that the 'more ancestral' NRC0s can initiate cell death by being activated by also more distantly related NRC0-S but that the 'younger' NRC0s are unable to be activated by more distantly related NRC0-Ss.

We thank the reviewer for the accurate summary and positive evaluation.

The phylogenetic analysis seem to be accurately performed and are very well documented (methods section). The interpretations are easily formulated and easy to comprehend. I very much like the mechanistic analysis to show the dependency of the NRC0-S partners of the NRC0s in the second part of the manuscript. This analyses nicely confirm their evolutionary findings and - as the authors also write in the discussion - opens up many possibilities to study how NRC-Ss may activate NRC-Hs.

We again thank the reviewer and we agree that the capacity to perform functional assays with the NRC0 system is exciting and adds another dimension to the study and that is sometimes lacking in purely phylogenetic papers.

I do have some minor issues/suggestions:

The authors write that some species (including N. benthamiana) have lost the NRC0 gene cluster and that this may be due to autoimmunity or cost trade-offs. Could it be possible that in the species where only NRC0s are found (without the linked NRC0-S genes that these NRC0s have no canonical MADA motif? Probably the authors know or have this information. If these NRC0s have a canonical MADA motif t would be great to see whether these are active (make the DV mutant and test for cell death in N. benthamiana). If these NRC0s are nonfunctional this result could confirm the hypothesis that they are costly to have and thus only inactive versions or no NRC0 is kept.

Thank you very much for your comment. In our analyses, we found that 10 plant species have NRC0, but not NRC0-S within 50 kb genetic distance. All of the NRC0 were predicted as MADA-CC-NLRs or MADA-like CC-NLRs (Supplemental Table S1). Although we have not yet determined whether the NRC0 carry cell death inducing activity, we hypothesize that the NRC0 may function together with NRC-S genes, which are located on chromosomes with over 50 kb distance from the NRC0 ortholog genes. Indeed, a very recent preprint by Goh et al. (https://www.biorxiv.org/content/10.1101/2023.10.25.563953v1) reported that lettuce NRC0 could trigger cell death response with NRC-S, whose genes are located on the chromosome 8 with 60 kb, 114 kb and 319 kb away from the NRC0 gene. Therefore, our computational pipeline to identify

NRC0-S partner genes is still technically limited in terms of genetic distance, quality of reference genome assembly and NLR gene annotation. We discussed this point below:

"Notably, five campanulids (*Lactuca sativa*, *Helianthus annuus*, *Mikania micrantha*, *Artemisia annua* and *Erigeron canadensis*) and four lamiids (*Olea europaea* subsp. *Europaea*, *Olea europaea* var. *sylvestris*, *Ipomoea triloba* and *Capsicum annuum*) have *NRC0* orthologs but no genetically linked *NRC0-S* encoded within 50 kb genetic distance. It is possible that *NRC0* and *NRC0-S* genes have been genetically dispersed in their genomes like in other sections of the NRC network. Indeed, as recently observed, *L. sativa* NRC0 functions with three NRC-S, *Ls124601*, *Ls123301* and *Ls124100*, whose genes are located on chromosome 8, 60 kb, 114 kb and 319 kb away from the *NRC0* gene, respectively (Goh et al., 2023)."

Is there any support for transcriptional co-regulation of the NRC0 and NRC0-S NLRs? They are clustered but not liked in a head-to-head orientation like the RPS4/RRS1 type of pairs. Would be interesting to have/see some data on this if available. This would also further support their functional relationship.

Thank you very much for your comment. We have added a transcriptome profile data of tomato NRC0 and NRC0-S genes in each leaf and root tissue. The new data is in Supplemental Figure S5A, which shows that SlNRC0 and SlNRC0-S are expressed in both of leaf and root tissues.

BN-PAGE experiment: Could it be possible that the aHA antibody is not working for BN-PAGEs? Did the authors try with a different tag or with swapped tags?

Thank you very much for your comment. The anti-HA antibody works well in the SDS-PAGE. One possibility for the lack of signal in the BN-PAGE blot is that the amount of protein expressed is too low to be detected in the native condition, where proteins run as smears instead of the single bands observed in SDS-PAGE gels. We often face similar technical issues with other NLRs in BN-PAGE experiments. In the future study, we will figure out the optimal conditions of NRC0-S for the BN-PAGE.

Reviewer #2 (Comments for the Author):

Plant immune receptors often exist as diverse series of alleles within a species, but their evolution at the genus, family, and superfamily scales is only beginning to be explored. The manuscript advances this goal by exploring the evolution of a specific helper NLR, describing early diverging orthologs of the NRC clade of NLR receptors. Extensive previous work on the NRC clade in Solanaceae has shown that helper NRCs (NRC-H) are genetically required for responses downstream of many sequence-related sensor NLRs termed "NRC-S". Here the authors show that the most early-diverging NRC-H, termed NRC0, has functional orthologs in outgroup Asterid species. NRC0 also frequently falls in a genetic cluster with sensor NLRs, and the clustered NLR pairs function similarly to the dispersed sensor-helper pairs described previously: autoactivating mutations in the sensors require the helper as shown using the N.benthamiana cell death assay and BN-PAGE analysis of helper NLR molecular complexes. Functional compatibility of NRC0 and NRC0-S orthologs is explored, but not extended combinations of NRC sensors and helpers (see below).

Thank you very much for your evaluation and comments.

Overall, the evolutionary analyses are excellent and presented very clearly. I especially like the visual representation of cluster evolution with a circos plot in Fig 2A, which nicely shows both lineage-specific and more ancient, conserved clusters. Importantly the work sets another benchmark for the

diversification rate of an ancient helper NLR including in specific motifs / subdomains. Alongside the separate case study of ZAR1 this could help provide predictive indicators for helper NLR status.

An ancient, stable sensor-helper pair, genetically sister to a diversified network, would be very interesting. Strong claims are made that the gene cluster is ancient, not just NRC0 itself (lines 39, 153, 332, 406). Unfortunately the claim regarding NRC0 cluster as an "ancestral state that predates [expansion of NRC network in lamiids]" is not well supported. Several lines of evidence refute the "ancestral state" hypothesis:

1. The hypothesis would predict that outgroup species would encode NRC0 homologs but not other NRC genes. In fact, the exact opposite case is true -- beet, tea, kiwi, and rhododendron encode other NRC-H/S genes, but not NRC0 (Fig 4, Fig 9).

Thank you very much for your comment. In this revision, we have included a Cornales species, Cornus florida, which has only NRC0, but no other NRC helper gene (Figure 4B). This finding supports our hypothesis that the NRC0 reflects an ancestral state that predates the massive expansion of the NRC network in the lamiid lineage of asterid plants. We discussed this point below:

"We used NLRtracker to annotate NLR genes from 32 asterid, one Caryophyllales and one Santalales species, and classified NRC-H and NRC-S genes based on phylogenetic analysis. We found NRC-H and NRC-S are drastically expanded in lamiids, compared to other plant orders in asterids (Figure 4B; Supplemental Table S3 and Supplemental File 12). In Cornales, Ericales, and campanulids, the number of NRC-H genes range from one to eight and that of NRC-S genes range from 0 to 31 across species in annotations and gene definitions used (Figure 4B; Supplemental Table S3 and Supplemental File 12). Notably, a Cornales species, *Cornus florida*, has two *NRC0* genes (XP_059649788.1 and XP_059645235.1), but no other NRC-H (Figure 4B). Although the *C. florida NRC0* genes are not genetically clustered with NRC-S genes within less than 50 kb genetic distance, eight and four NRC-S genes are located on the same scaffolds with XP_059649788.1 and XP_059645235.1, respectively (Supplemental Figure S3)."

Sugar beet NRC helper gene is in the outgroup of NRC0 and other NRC subclades. Therefore, we think that the NRC0 gene cluster probably originated from a common ancestral NLR pair that might be shared with the Caryophyllales NRC gene pair.

2. The hypothesis would predict that not only NRC0 but also the paired sensors (NRC0-S) would also be related by orthology, thus NRC0-S would form an ancient, outgroup clade sister to NRC-S genes. Instead, phylogenetically distant NRC0-S genes exist at the NRC0 locus (Fig 3), suggesting repeated acquisition of diverse sensors at the locus. While still interesting, this suggests that NRC0 is just acting like the other well-characterized NRCs, it just happens to be the oldest MADA-containing helper. Is there anything special about the NRC0-S genes which happen to function via NRC0?

Thank you very much for your comment. We agree with the reviewer comment that unlike NRC0, NRC0-S are distantly distributed in the NRC-S clade, suggesting that NRC0-S genes are evolving faster or are repeatedly acquired at the locus. Due to sequence diversity in NRC-S subclades, we faced a technical difficulty of detailed phylogenetic classification of NRC-S genes with robust bootstrap supports. We removed and toned down about the evolution of NRC0-S gene throughout the manuscript.

3. The NRC0 locus is not always a cluster; it is a single gene without NRC0-S homologs in the outgroup lamiids and campanuiids. Since the clustered sensor NLR is not stable (point 2 above), the ancestral state was probably a locus encoding only NRC0, not a cluster.

Thank you very much for your comment. We hypothesize that the NRC0 may function together with NRC-S genes, which are located on chromosomes with over 50 kb distance from the NRC0 ortholog genes. Indeed, a very recent preprint by Goh et al. (https://www.biorxiv.org/content/10.1101/2023.10.25.563953v1) reported that lettuce NRC0 could trigger cell death response with NRC-S, which are located on the chromosome 8 with 60 kb, 114 kb and 319 kb away from the NRC0 gene. Therefore, our computational pipeline to identify NRC0-S partner genes is still technically limited in terms of genetic distance due to the varying quality of reference genome assembly and NLR gene annotation. We toned down our conclusion throughout the manuscript with a sentence "the NRC0 sensor/helper gene cluster may reflect an ancestral state that predates the massive expansion of the NRC network in the lamiid lineage of asterid plants".

Overall, I agree that NRC0 is an ancient, slowly evolving NRC-H. While still quite interesting, its function should be shown in the context of the broader NRC network. The critical missing experiments are A) whether NRC0 can enable function of NRC-S genes besides those encoded at the locus itself (such as Rx Bs2 or Prf), and B) whether autoactive NRC0-S function can be enabled by other NRCs. (NRC4 is tested in Fig 7 but not other genes.)

If NRC0 facilitates the activity of non-clustered sensor NLRs, not just NRC0-S genes, the major claim of the manuscript should be revised: NRC0 would be an example of a helper with a stable function and no evidence of diversification, but it would still function within a diverse network, not function as a separate sub-cluster.

Thank you very much for your comment. We conducted the suggested experiments added in Supplemental Figures S4 and S5. Please see response to editors above.

Other comments:

- Synteny analysis should be performed in order to confirm loss of NRC0 locus in the Ericales. Genes bordering NRC0 are expected to show synteny with Ericales and related asterids, but with a synteny gap at the NRC0 locus in the Ericales. Without this positive evidence for an NRC0 deletion it is difficult to conclude the absence of NRC0 solely based on BLAST analysis.

Thank you very much for your comment. We tried synteny analysis, but even if using closely related asterid species genome, we failed to observe clear synteny surrounding the NRC0 ortholog genes. Therefore, we think it is difficult to conclude about the loss of NRC0 in Ericales based on the synteny analysis. We removed "absent in Ericales" and toned down this point throughout the manuscript.

- inset panel in Fig 1A and Suppl Fig S1: it is important to show where the NRC clade is rooted to show that NRC0 are the outgroup of all other NRCs. Could the supplemental fig show the root, or an even further outgroup? The main figure legend should refer to the suppl. fig for rooting.

Thank you very much for your comment. We revised the figures as you suggested.

- Fig 9: The NRC0 loci are not identical, as indicated in the figure. The lamiids subpanel is also confusing to me. Do the different lines indicate different lamiid species? Or does the panel indicate a single species with 24 NRC loci?

Thank you very much for your comment. Figure 9 is a schematic representation of NRC0 gene cluster and genetically dispersed networks, and does not refer to actual NRC loci in each species. To avoid confusion, we simplified the model by removing scheme of crops.

Reviewer #3 (Comments for the Author):

The manuscript by Sakai et al. reports on the identification of a NRC gene pair including a helper immune receptor termed NRC0 and a cognate sensor NRC0-S that are broadly conserved across asterid plants. The ancestral NRC0 - NRC0-S pair is proposed to be the ancestral state of diversified helper-sensor modules/network found in modern asterids because this pair is the most conserved identified in asterid genomes and several modern NRC0 - NRC0-S pairs show similar signaling ability.

Research on plant immune receptor is a very active field. The architecture and evolution of immune receptor netwoks adressed in this study is timely and relevant. The analyses and experiment are carefully conducted and reported clearly. The methods and discussion sections should be completed/revised and some claims and conclusions should be nuanced as detailled hereafter.

Thank you very much for your evaluation and comments.

Major comments:

1. Genome mining and phylogenetic analyses identified NRC0 as the only NRC gene conserved in 4 out of 6 asterid species. Some details would be useful to provide in the M&M for this section:
- how was the substitution model selected?

Thank you very much for your comment. We have explained this in the materials and methods section.

- how many informative sites were selected from the alignments?

Thank you very much for your comment. We have Supplemental Files 2, 4, 6, 8 and 11 of multiple sequence alignments for readers to reproduce our phylogenetic analyses.

- are the trees rooted, if yes which sequence was used as outgroup?

Thank you very much for your comment. We explained rooted or unrooted tree in figure legends, and we described outgroup information for the rooted trees.

2. Experiments in Nicotiana benthamiana indicate that NRC0 - NRC0-S pairs form functional signaling modules conserved in serveral asterids. NRC0 from carrot and coffe signal with NRC0-S from carrot and coffee but not orthologs from sweet wild potato and tomato, suggesting some degree of co-evolution. If NRC0 was the closest descendent of the ancestral NRC network, I would expect the carrot and coffee alleles, from species that diverged earlier, to show a lower degree of specialization regarding the NRC0 sensors they can signal with. What does the observed specialization pattern tell us about the proposed ancestral NRC network?

Thank you very much for your comment. Based on our cell death assays, we hypothesized that the 'more ancestral' NRC0s are activated by more distantly related NRC0-S, but that the 'younger' NRC0s are unable to be activated by more distantly related NRC0-Ss. However, our knowledge about ancestral NRC sensor – helper compatibility and co-evolution is still limited. In the future, we will narrow dawn and study how functional specificity between NRC0 and NRC0-S is determined.

3. Throughout the manuscript, some claims regarding the NRC network evolution extrapolate on the actual evidence. They should be further discussed and nuanced (or additional data provided if available):

"functionally conserved NRC0 gene cluster" > the signaling function downstream of NRC0-S is indeed conserved as shown in this work. However it is not known whether the recognition range of the NRC0 - NRC0-S module is conserved, as mentioned in the manuscript's discussion.

Thank you very much for your comment. As you mentioned, we have not yet revealed what kind of effectors NRC0-S/NRC0 recognizes across asterids. This is indeed a fascinating question, what is this locus recognizing that makes it so conserved. In the future, we will work on this point using NRC0 and NRC0-S pairs from different plant species.

"the NRC0 sensor-helper gene cluster reflects the ancestral state of the NRC network":

- NRC gene deletion might be frequent, as observed for NRC0 in Ericales, so that the Solanales/asterid ancestor species might have included a more complex network than can be inferred based on conserved genes alone.

Indeed, NRC gene deletions may have occurred in Ericales as well as in other Solanales/asterid ancestor species, and thus there may have been a more complex ancestral network architecture. However, based on the analyses performed here, including the newly added Cornus florida data, we believe that the NRC0 defined here, rather than other NRC network, is more likely to reflect the ancestral state of the NRC network. Some other NRC networks of asterid species were identified and analyzed in Goh et al (2023).

- Whether other (clustered) NRCs derive from the NRC0 - NRC0-S pair remain to be established. The phylogenetic tree from figure 4A seem to suggest that since the split with Cornales, NRC0 and other NRC-H already formed two distinct clades.

Thank you very much for your comment. In this revision, we have included a Cornales species, Cornus florida, which has only NRC0, but not other NRC helper gene (Figure 4B). This finding supports our hypothesis that the NRC0 reflects an ancestral state that predates the massive expansion of the NRC network in the lamiid lineage of asterid plants. Please note that we carefully use the term “reflects” an ancestral state of the expanded network to leave room for alternative scenarios.

- Can NRC0 signal (trigger cell death) with sensors outside the NRC0-S clade? This would provide support for NRC0 function pre-dating the divergence of the NRC0 clade.

Thank you very much for your comment. We conducted additional experiments using other NRC helpers and sensors, as shown in Supplemental Figures S4 and S5. Please see response to editors above.

Alternative ancestral NRC network architectures might be worth discussing in the light of these questions/information.

Thank you very much for your comment. By including new information of NRC genes in one Cornales, Cornus florida, and one Santalales species, Malania oleifera, we discussed more the evolution of NRC networks in asterids and emergence of NRC family gene.

Dear Dr. Hiroaki Adachi:

We have received reviews of your manuscript entitled "The NRC0 gene cluster of sensor and helper NLR immune receptors is functionally conserved across asterid plants." On the basis of the advice received, the board of reviewing editors would like to accept your manuscript for publication in The Plant Cell. This acceptance is contingent on revision based on the comments of our reviewers. In particular, please consider the following:

Plase edit the discussion to highlight alternative hypotheses regarding the ancestral state, following comments from reviewers 2 and 3.

Please highlight all changes and include a detailed annotation of changes of the text, with line numbers, and noting your responses to the comments.

To submit your revised manuscript, click:

Link Not Available

If you have any questions about the revision submission procedures, please contact the Editorial Office Staff (tpc-submissions@aspb.org). If you cannot return the revised manuscript within 30 days, please let us know. Otherwise, we will assume that you have elected not to revise the manuscript and withdraw it.

Thank you very much for the privilege of reviewing this work. I look forward to receiving the next version.

Sincerely,

The Plant Cell Editorial Board

(Please note that due to a potential conflict of interest, EIC Blake Meyers did not participate in evaluating this manuscript).

Reviewer #1 (Comments for the Author):

This revision of the mansucript is a responding to all the comments/suggestions and critics of the reviewers, including mine. The authors responses are justafieble, convincing and helpful. I like the inclusion of the experiment suggested by reviewer 2 (NRC0-H activation by known NRC-dependent functionaly characterized NLRs). The revised version has improved from the authors work and the reviewewrs suggestions. I do not have any further concerns. It is a great study and thetext was a pleasure to read. Thanks.

Reviewer #2 (Comments for the Author):

The revised manuscript is stronger. First, an additional outgroup species Cornus florida (dogwood) is shown to have NRC0, further supporting the claim that it is an ancient outgroup to the rest of the NRC-H genes but has not expanded in non-lamiid lineages. It is still strange that beet has lost NRC0 -- and I guess there may someday be a Caryophyllales genome which has still retained its NRC0?

Second, new HR assays support the concept of NRC0 - NRC0-S specificity by testing SlNRC0 and SlNRC0-S against other sensors and helpers. Thus the other clustered NRC0 - NRC0-S genes are likely to be functionally related. It would be very interesting in future studies to determine how diverse NRC0-S genes convergently evolved the ability to function with a conserved NRC0. (for example, DcNRC0-S vs SlNRC0-S are very divergent, Fig 2).

The paper's claim of the ancestral state being a cluster (lines 159, 449, 463) is still in my opinion not fully supported based on point #2 from my previous review, but this is a matter of interpretation and does not detract from a very strong paper.

Reviewer #3 (Comments for the Author):

Overall I found that the additional results and edits to the text that the authors implemented in the revised manuscript adressed reviewers comments appropriately.

I have a few minor suggestions to further clarify the text:

. The authors refer to NRC0 as reflecting "an ancestral state" for the networks existing in lamiids. It is an important but very stuble nuance to "the ancestral state" to which readers might be more accustomed, and therefore may easily be missed. I would therefore appreciate if somewhere in the discussion section the authors would explicitely mention alternative ancestral states that would be compatible with their results and mention remaining questions regarding the actual ancestral state of the NRC network (eg. is NRC0 the closest relative to the ancestor from which current lamiid NRCs derive?; or if several ancestral states are likely, what form of convergence occured)

. The authors refer to NRC0 as "functionally conserved" although they acknowledge that the extent of this functional conservation is not fully determined yet. While this shortcut it convenient and probably appropriate for the title and abstract, it might be useful to state more explicitely what is meant (and not meant) by "functionally conserved" in the last paragraph of the discussion where this notion appears one last time.

abstract L38: better use "provide support for" instead of "validated"

Please also note the following:

-The Plant Cell now requires authors to complete and submit an author revisions checklist upon submission of a revised manuscript. The aim of the checklist is to aid authors in preparing a high-quality manuscript, facilitate the review and assessment of revised manuscripts, and help to ensure that journal standards are maintained across the board. If your manuscript is accepted, the completed checklist will be published as supplemental material attached to the article online. Please download a copy of the checklist (pdf fillable form) at this link, for submission with your revised manuscript: https://tpc.msubmit.net/html/Author_Revisions_Checklist.pdf.

-Supplemental materials should be restricted to large datasets and tables, presentation of replicates, and validation of reagents, methods, or genotypes. Any data that are used to support the major claims must be in the main manuscript. Supplemental figure legends must indicate what figure in the main manuscript is supported by the supplemental data presented. Please justify how each of the supplemental figures meet the criteria.

-Sampling methods and the nature of "biological replicates" should be described precisely (i.e. different plants, parts of plants, pooled tissue, independent pools of tissue, sampled at different times, etc.), along with a clear description of and rationale for any statistical analyses conducted. The reader should know exactly what was sampled; what forms the basis of the calculation of any means and statistical parameters reported. This is also necessary to ensure that proper statistical analysis was conducted.

Dear Dr. Pierre-Marc Delaux and Dr. Roger Innes,

Thank you very much for your evaluation of our manuscript " The *NRC0* gene cluster of sensor and helper NLR immune receptors is functionally conserved across asterid plants " and for acceptance of revised manuscript as a Large-Scale Biology article at The Plant Cell.

We appreciate the feedback of the independent reviewers. We have endeavored in this revision to address as much as possible the pertinent points they raised. Please see the point-by-point 'Response to Reviewers' attached to this file.

We hope that we have addressed many of the issues that were raised, and we look forward to your response.

Best regards,

Hiroaki Adachi

Dear Dr. Hiroaki Adachi:

We have received reviews of your manuscript entitled "The NRC0 gene cluster of sensor and helper NLR immune receptors is functionally conserved across asterid plants." On the basis of the advice received, the board of reviewing editors would like to accept your manuscript for publication in The Plant Cell. This acceptance is contingent on revision based on the comments of our reviewers. In particular, please consider the following:

Thank you very much for your evaluation and positive comments.

Please edit the discussion to highlight alternative hypotheses regarding the ancestral state, following comments from reviewers 2 and 3.

Thank you very much for your comment. We edited the discussion section by addressing suggestions from the reviewers. Please see our responses below.

Please highlight all changes and include a detailed annotation of changes of the text, with line numbers, and noting your responses to the comments.

Thank you very much for the privilege of reviewing this work. I look forward to receiving the next version.

Reviewer #1 (Comments for the Author):

This revision of the manusucript is a responding to all the comments/suggestions and critics of the reviewers, including mine. The authors responses are justafieble, convincing and helpful. I like the inclusion of the experiment suggested by reviewer 2 (NRC0-H activation by known NRC-dependent functionaly characterized NLRs). The revised version has improved from the authors work and the reviewewrs suggestions. I do not have any further concerns. It is a great study and thetext was a pleasure to read. Thanks.

We thank the reviewer for your evaluation and positive comments.

Reviewer #2 (Comments for the Author):

The revised manuscript is stronger. First, an additional outgroup species Cornus florida (dogwood) is shown to have NRC0, further supporting the claim that it is an ancient outgroup to the rest of the NRC-H genes but has not expanded in non-lamiid lineages. It is still strange that beet has lost NRC0 -- and I guess there may someday be a Caryophyllales genome which has still retained its NRC0?

Thank you very much for your evaluation and comments. As described in Figure 9, we hypothesized the NRC0 gene emerged after Caryophyllales and asterid lineages split, because we have not yet found the NRC0 ortholog gene from currently available Caryophyllales genomes. It is likely that the NRC0 gene probably originated from a common ancestral NRC that might be shared with the Caryophyllales NRC gene pair, as mentioned in discussion (Line 454 to 455).

Second, new HR assays support the concept of NRC0 - NRC0-S specificity by testing SlNRC0 and SlNRC0-S against other sensors and helpers. Thus the other clustered NRC0 - NRC0-S genes are likely to be functionally related. It would be very interesting in future studies to determine how diverse NRC0-S genes convergently evolved the ability to function with a conserved NRC0. (for example, DcNRC0-S vs SlNRC0-S are very divergent, Fig 2).

Thank you very much for your comment. We agree with your opinion that elucidation of the functional specificity between NRC0 and NRC0-S is an interesting research topic for the future study.

The paper's claim of the ancestral state being a cluster (lines 159, 449, 463) is still in my opinion not fully supported based on point #2 from my previous review, but this is a matter of interpretation and does not detract from a very strong paper.

Thank you very much for your comment. As the reviewer suggested, we added hypotheses that NRC0-S genes are fast-evolving or have been repeatedly acquired at the NRC0 locus. Please see discussion section (Line 484-489).

Reviewer #3 (Comments for the Author):

Overall I found that the additional results and edits to the text that the authors implemented in the revised manuscript adressed reviewers comments appropriately.

Thank you very much for the positive comment.

I have a few minor suggestions to further clarify the text:
. The authors refer to NRC0 as reflecting "an ancestral state" for the networks existing in lamiids. It is an important but very stuble nuance to "the ancestral state" to which readers might be more accustomed, and therefore may easily be missed. I would therefore appreciate if somewhere in the discussion section the authors would explicitely mention alternative ancestral states that would be compatible with their results and mention remaining questions regarding the actual ancestral state of the NRC network (eg. is NRC0 the closest relative to the ancestor from which current lamiid NRCs derive?; or if several ancestral states are likely, what form of convergence occured)

Thank you very much for your comment. We edited the discussion section with having an alternative hypothesis that the NRC networks may have originated from other ancestral NRC gene pair(s). Please see Line 463-465.

. The authors refer to NRC0 as "functionally conserved" although they acknowledge that the extent of this functional conservation is not fully determined yet. While this shortcut it convenient and probably appropriate for the title and abstract, it might be useful to state more

explicitely what is meant (and not meant) by "functionally conserved" in the last paragraph of the discussion where this notion appears one last time.

Thank you very much for your comment. We rephrased “a functionally conserved NLR gene cluster” to “an NLR gene cluster, where the NRC helper is genetically and functionally linked to sensor NLR” in the last paragraph of the discussion (line 536-537).

abstract L38: better use "provide support for" instead of "validated"

We edited this.

Dear Dr. Hiroaki Adachi:

We are pleased to inform you that your paper entitled "The NRC0 gene cluster of sensor and helper NLR immune receptors is functionally conserved across asterid plants" has been accepted for publication in The Plant Cell, pending a final editorial review by a science editor. At this stage, your manuscript will be evaluated by a science editor with respect to its presentation of scientific content, compliance with journal policies, and presentation for a broad readership. The Plant Cell has contracted with Plant Editors (planteditors.com) to provide this service to our authors, and you will soon receive additional information on this process.

Please note that each author needs to link their ORCID identifier to their account in the system before your manuscript can be published. If any authors do not have an ORCID linked to their account, they will receive a message with a link to complete this task. Please ensure that ALL of your coauthors have completed this task as soon as possible.

ASPB offers an OPEN option that allows authors to have their online articles available for free to all users immediately upon publication. For more information about the ASPB OPEN option, refer to the Final Submission Checklist Form.

The Plant Cell and The Arabidopsis Information Resource (TAIR) are collaborating to collect functional annotation data about Arabidopsis genes from authors. This includes information about the gene's molecular function (e.g., kinase activity, ATP synthetase activity), the biological process/es it is involved in (e.g., endosperm development, threonine biosynthesis), its subcellular location (e.g., nucleus, ER), anatomical or developmental expression pattern (e.g., leaf, ovule, flower stage 10, seedling stage), or its partner in a protein-protein interaction (e.g., AT1G01010 interacts with AT1G01020).

If your paper contains results falling into one or more of these categories for Arabidopsis genes, we request that you now submit these data for inclusion in TAIR by filling in the form provided at the following URL: https://www.arabidopsis.org/doc/submit/functional_annotation/123. If you need further clarification on what types of data can be submitted please contact curator@arabidopsis.org.

Finally, we encourage your submission of artwork for the journal cover. Monthly issues will have an online cover image and selected covers will be used for posters, other promotional items, and "wallpaper" for mobile devices. For more information, refer to Cover Submission in the Instructions for Authors https://tpc.msubmit.net/cgi-bin/main.plex?form_type=display_auth_instructions.

We look forward to seeing your paper published.

Sincerely,

The Plant Cell Editorial Board

(Please note that due to a potential conflict of interest, EIC Blake Meyers did not participate in evaluating this manuscript).

=====
IMPORTANT REMINDER: PEER REVIEW REPORTS
=====

If you opted to publish a peer review report along with your article during the original submission process, it will be prepared by the editorial staff and publicly posted with your manuscript, inside the zip file that contains any other supplemental material. As a reminder, the peer review report is a public record of all comments from editors and reviewers, as well as your prior responses, as you received them in the decision letters for each draft of your manuscript. If you agreed to publish this report and have changed your mind, or are not sure if you selected this option, please contact the editorial office as soon as possible before signing the license agreement from our publisher.

----FOR ASPB OFFICE USE ONLY (DO NOT EDIT)----
MSID: 37020
Corresponding Author: Dr. Adachi
